# The identification of adenylyl cyclase modulators as potential receptors for 6-nitrodopamine in human-induced pluripotent stem cell (hiPSC)-derived cardiomyocytes and their relevance in heart inotropism

**DOI:** 10.3389/fphar.2025.1597035

**Published:** 2025-08-11

**Authors:** Irene Cipollone, Vittoria Monaco, José Britto-Júnior, Antonio Tiago Lima, Edson Antunes, Andre Sampaio Pupo, Ilaria Iacobucci, Flora Cozzolino, Maria Monti, Silvia Parisi, Giuseppina Divisato, Emanuela Cascone, Alfonso De Simone, Angela Corvino, Ferdinando Fiorino, Francesco Frecentese, Vincenzo Santagada, Beatrice Severino, Rosa Sparaco, Pierfrancesco Cinque, Stefania Vertuccio, Giuseppe Caliendo, Gilberto De Nucci

**Affiliations:** ^1^ Department of Chemical Sciences, University of Naples Federico II, Naples, Italy; ^2^ CEINGE Biotecnologie Avanzate “Franco Salvatore”, Naples, Italy; ^3^ Department of Pharmacology, Faculty of Medical Sciences, State University of Campinas (UNICAMP), São Paulo, Brazil; ^4^ Department of Biophysics and Pharmacology, Institute of Biosciences of Botucatu, São Paulo State University (UNESP), Botucatu, Brazil; ^5^ Department of Molecular Medicine and Medical Biotechnology, University of Naples Federico II, Naples, Italy; ^6^ Department of Pharmacy, School of Medicine, University of Naples Federico II, Naples, Italy; ^7^ Department of Pharmacology, Institute of Biomedical Sciences, University of São Paulo (ICB-USP), São Paulo, Brazil

**Keywords:** dopamine, adrenaline, nitro-catecholamine, stromal interaction molecule 1, cyclase-associated proteins

## Abstract

6-Nitrodopamine (6-ND) has potent positive chronotropic and inotropic effects. At a very low dose, i.e., 10 fM, it causes potentiation of the positive chronotropic effects induced by catecholamines in the rat atria, indicating a distinct mechanism of action. Cyclase-associated proteins (CAP-1 and CAP-2) are potential receptors for 6-ND in human cardiomyocytes. Since cyclic 3′,5′-cyclic adenosine monophosphate (cAMP) plays a fundamental role in the positive inotropic effects of classical catecholamines, it was further investigated whether 6-ND potentiates the positive inotropic effects induced by classical catecholamines in the rat isolated perfused heart. Human-induced pluripotent stem cell (hiPSC)-derived cardiomyocytes were harvested and lysed, and following appropriate separation procedures, membrane proteins were incubated with 6-ND-derivatized agarose, centrifuged, and the proteins retained in the agarose eluted with 6-ND (1 mM). The proteins isolated from the chemical pulldown assay were fractionated by SDS-PAGE, the bands were cut and hydrolyzed *in situ* with trypsin, and then separated and sequenced. A total of 817 proteins were generated, and following screening using UniProt “Retrieve/ID Mapping” function and Gene Ontology cellular component category, 124 proteins were identified as membrane proteins. These experiments identified three proteins that modulate adenylyl cyclase (AC) activity (CAP-1, CAP-2, and STIM1), which are compatible with the pharmacological findings reported for 6-ND in the rat heart. As expected, 6-ND strongly potentiates the inotropic effect induced by noradrenaline in Langendorff’s preparation. In conclusion, 6-ND-induced potentiation of catecholamine-induced chronotropic and inotropic effects is due to the modulation of adenylyl cyclase activity, probably via direct interactions with CAP-1 and CAP-2.

## 1 Introduction

6-Nitrodopamine (6-ND) is a novel endogenous catecholamine that exerts potent and long-lasting positive chronotropic and inotropic responses in the isolated rat heart (Britto-Júnior et al., 2022; 2023a; Zatz and De Nucci, 2024). In rat isolated right atria, 6-ND at a concentration as low as 10 fM markedly potentiates the positive chronotropic effects induced by the classical catecholamines dopamine, noradrenaline, and adrenaline (Britto-Júnior et al., 2023b). Interestingly, the low concentration (100 nM) of selective β1-adrenoceptor antagonists atenolol, betaxolol, and metoprolol significantly reduced both basal atrial rates and 6-ND-induced positive chronotropism (Britto-Júnior et al., 2022). This result implies that these drugs act as 6-ND receptor antagonists (Britto-Júnior et al., 2023b). More recently, 4-nitropropanolol (Sparaco et al., 2022) has been identified as a more selective 6-ND receptor antagonist in the rat isolated right atrium (Oliveira et al., 2024b); however, the receptor of 6-ND is yet to be identified.

Human-induced pluripotent stem cell (hiPSC)-derived cardiomyocytes (Di Baldassarre et al., 2018) reproduce the molecular mechanisms involved in heart disorders such as long QT syndrome (Keller et al., 2005), catecholamine polymorphic ventricular tachycardia (Itzhaki et al., 2012), and Brugada syndrome (Davis et al., 2012), thus offering a suitable translational model for investigating the expression of 6-ND receptors. Therefore, we employed hiPSC-derived cardiomyocytes to explore the potential targets for 6-ND, using a chemical pulldown approach, in which 6-ND was immobilized on agarose beads and incubated with cardiomyocyte membrane extracts. The purified proteins were then fractionated by SDS-PAGE; the bands were cut and hydrolyzed with trypsin, and the peptide mixtures were separated through UPLC and analyzed using mass spectrometry. Protein identification was carried out using the MaxQuant search-engine to query the UniProt *Homo Sapiens* database. In the competitive assay, 4-nitropropranolol, here referred to as a selective 6-ND antagonist, was added to the protein extract, and the proteins retained on the beads were subtracted from that obtained in the absence of 4-nitropropranolol. Three proteins that modulate the adenylyl cyclase (AC) activity were identified. Because cyclic 3′,5′-cyclic adenosine monophosphate (cAMP) plays a fundamental role in the positive inotropic effects of classical catecholamines (Guellich et al., 2014), we demonstrated the synergistic effects of 6-ND with noradrenaline on the positive inotropic response by using the Langendorff isolated heart perfusion model.

## 2 Materials and methods

### 2.1 Chemical pulldown assay and protein separation

**FIGURE 1:**
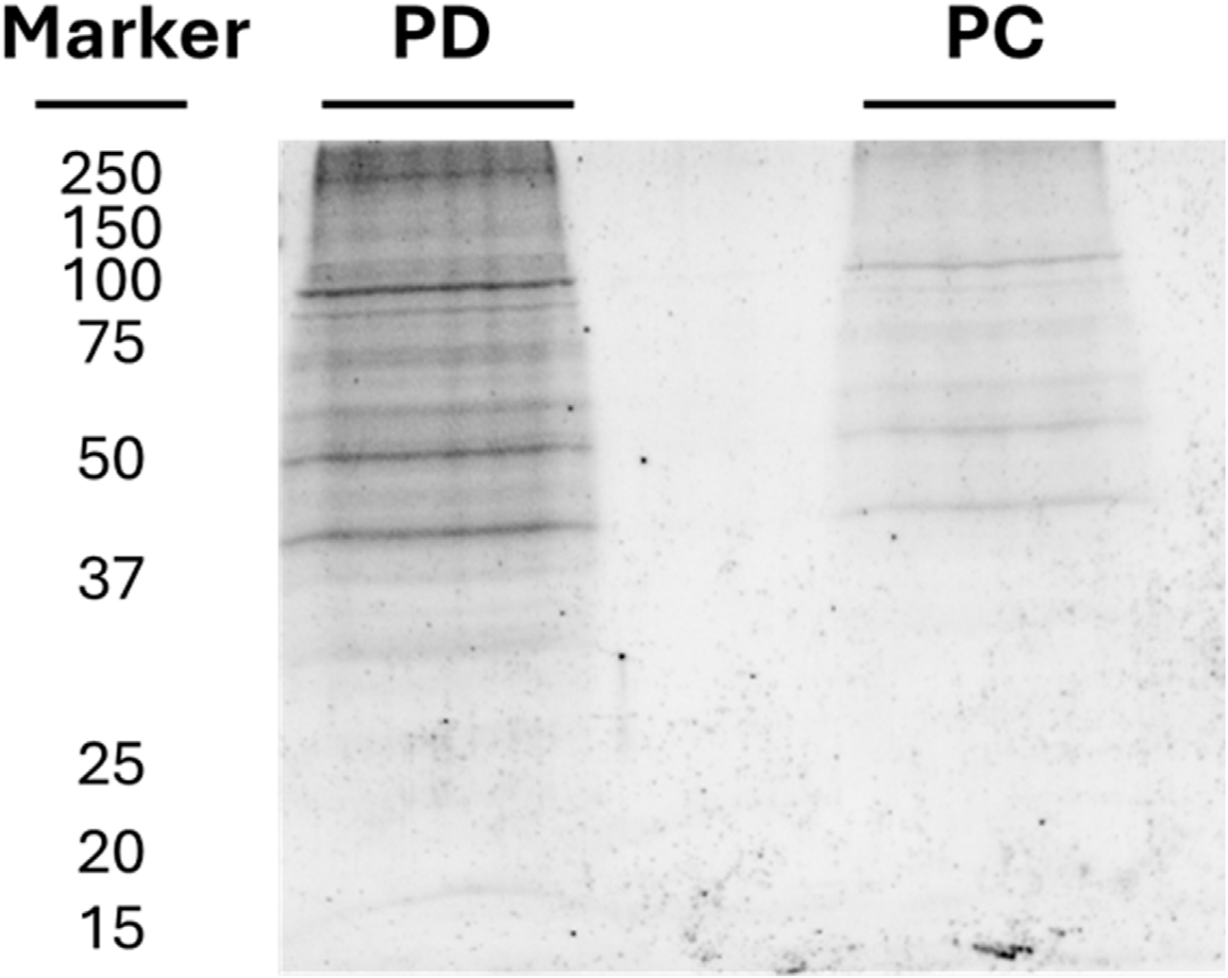
SDS-PAGE of proteins eluted from the chemical pulldown (lane PD) and from the pre-clearing (lane PC). The molecular weight markers are also reported.

Actively beating hiPSC-derived cardiomyocytes (iPS-DF19-9-7T, WiCell Research Institute, Madison, Unites States) were harvested and lysed with a specific membrane protein enrichment protocol. Cardiomyocytes were treated using a Mem-PER™ Plus Membrane Protein Extraction kit (ThermoFisher Scientific, Waltham, Massachusetts, United States), according to the manufacturer’s protocol to enrich the protein membrane protein extract. In brief, 50 × 10^6^ cardiomyocytes were added to 7.5 mL of permeabilization buffer, vortexed, and incubated for 10 min at 4°C with constant mixing. The supernatant, comprising cytosolic proteins, was collected by centrifuging for 15 min at 21,000 g. Solubilization buffer (5 mL) was added to the pellet, vortexed, and incubated for 30 min at 4°C with constant mixing. The supernatant, comprising the membrane proteins, was collected by centrifugation for 15 min at 21,000 *g*. The quantification of the extracted proteins was performed using the Pierce 660 nm assay (ThermoFisher Scientific, Waltham, Massachusetts, United States). The efficiency of the fractionation procedure was analyzed by Western blot assays, monitoring specific markers for each cell compartment (GAPDH for cytosol and caveolin-1 for membrane) with specific antibodies (Figure 1).

### 2.2 Chemical pulldown assay

**FIGURE 2:**
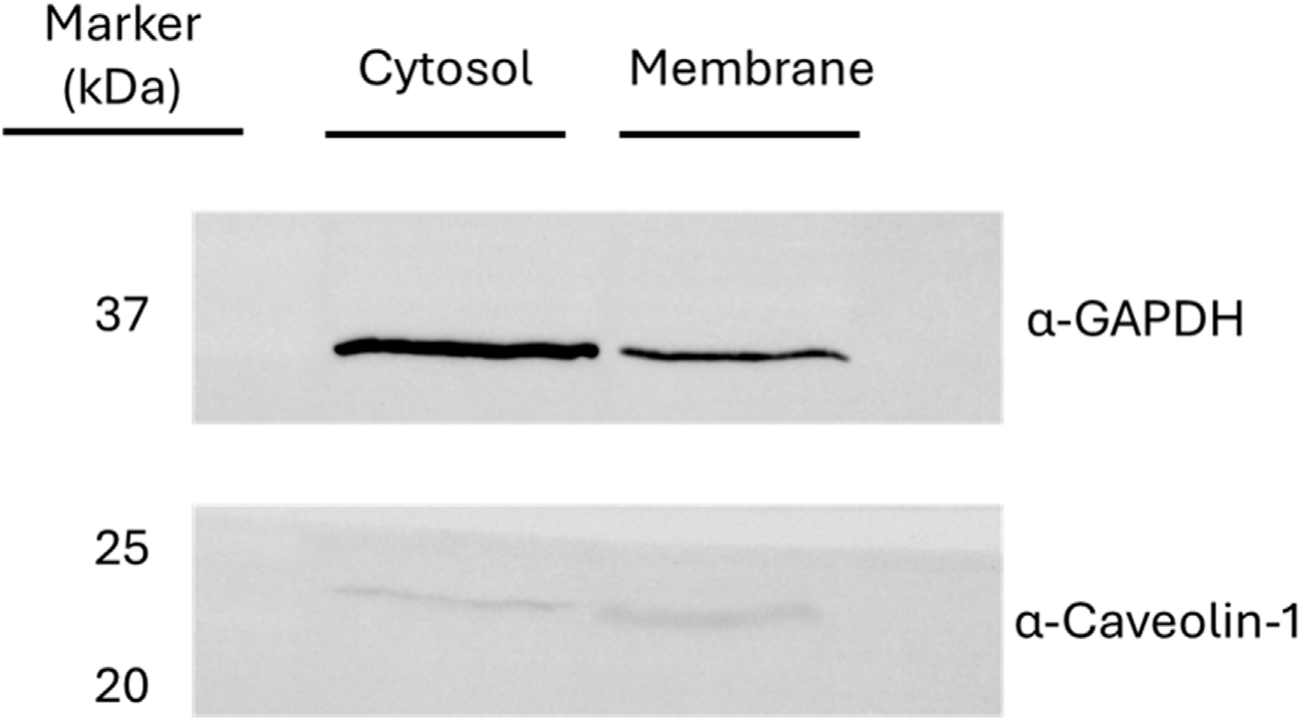
Western blot assays for the verification of the fractionated lysis. The presence of glyceraldehyde-3-phosphate dehydrogenase (GAPDH) and caveolin-1 was monitored as markers of cytosolic and membrane fraction, respectively.

For the pre-clearing step, 1.5 mg of the membrane extract was incubated (2 h) with 200 μL of naked PureCube Carboxy Agarose beads (Cube Biotech, Monheim, Germany) at 4°C to adsorb the protein background. The beads were centrifuged at 240 *g* for 2 min, and the supernatant was collected and then incubated with 200 μL of the resin derivatized with 6-ND overnight at 4°C. The naked agarose was washed with membrane extract buffer provided by the Mem-PER™ kit, and the proteins retained on the naked agarose were eluted with a solution of 1 mM of 6-ND. The eluted proteins were fractionated by SDS-PAGE. The gel was stained with GelCode™ Blue Safe Protein Stain (Thermo Fisher Scientific, Waltham, MA, United States) and destained with Milli-Q water (PC lane in Figure 2).

The supernatant was incubated overnight with the 6-ND-derivatized agarose and then centrifuged, and the pellet was washed with membrane extract buffer provided by the Mem-PER™ kit. The proteins retained on the 6-ND-derivatized agarose were eluted with a solution of 1 mM of 6-ND (PD lane in Figure 2).

In the competitive assay, 1 mM of 4-nitropropanolol was added to the membrane protein extract collected after the 6-ND pulldown assay and then incubated with the resin derivatized with 6-ND overnight at 4°C. The supernatant was removed, and the resin was washed with membrane extraction buffer provided by the Mem-PER™ kit. The proteins retained on the resin were eluted with a solution of 1 mM of 6-ND.

To define the interactors shared between 4-nitropropanolol and 6-ND, we identified the proteins retained on the beads in the competitor assay and subtracted this list from that obtained in the absence of the competitor, as described above.

### 2.3 Protein separation and identification

**FIGURE 3:**
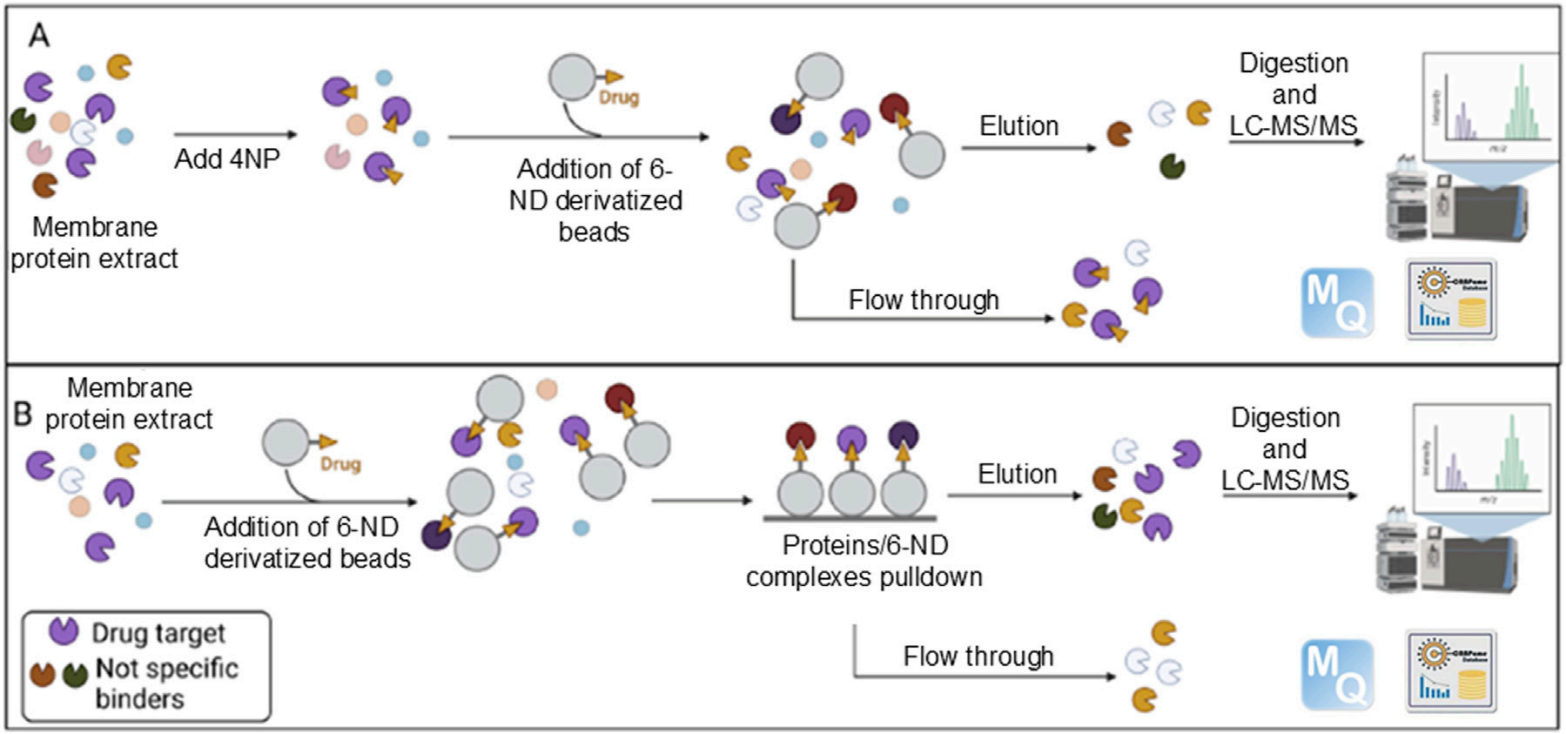
Graphical summary of the experimental strategy. Enriched membrane proteins were incubated with beads covalently coupled to 6-ND, either in the presence **(A)** or in the absence **(B)** of 4-nitropropranolol (4NP). Proteins binding to 6-ND and retained on the beads were subsequently eluted and identified through LC-MS/MS analysis. Adapted form Iacobucci et al. (2023), licensed under CC BY 4.0.

The proteins derived from chemical pulldown were fractionated by SDS-PAGE. The gel was stained with GelCode™ Blue Safe Protein Stain (Thermo Fisher Scientific, Waltham, MA, United States) and destained with Milli-Q water. A total of nine bands were cut and *in situ-*hydrolyzed by trypsin (Iaconis et al., 2017). Peptide mixtures were extracted in 0.2% HCOOH and ACN and vacuum-dried using a SpeedVac System (Thermo Fisher Scientific). Peptide mixtures from the hydrolyzed gel bands were analyzed on an Orbitrap Exploris 240 instrument equipped with a Nanospray Flex ion source and coupled with a Vanquish Neo nanoUPLC system. Samples were fractionated using a C18 capillary reverse-phase column (150 mm, 75, 2 μm 100 Å) at a flow rate of 250 nL/min. A linear gradient of eluent B (0.2% formic acid in 95% acetonitrile) in A (0.2% formic acid and 2% acetonitrile in LC-MS grade water) was used from 2% to 90% in 77 min. The MS/MS method, based on a data-dependent acquisition (DDA) mode, recorded a single full-scan spectrum in the 375–1,200 m/z range, followed by fragmentation spectra of the top 20 ions (MS/MS scan) selected according to the intensity and charge state (+2, +3, and multi-charges), with a dynamic exclusion time of 40 s. Protein identification was carried out using MaxQuant software (v.1.5.2.8), with the UniProt *Homo Sapiens* database, as previously described (Palinski et al., 2021). A diagram illustrating the above-described steps is presented in Figure 3.

Nonspecific contaminants were also removed for comparison using the Contaminant Repository for Affinity Purification (CRAPome) 2.0 web tool (https://reprint-apms.org). Contaminants defined as proteins reported in at least 50% of similar experiments were discarded from the initial lists (Iacobucci et al., 2022).

### 2.4 Functional clustering analysis

Cell compartment enrichment analysis was carried out using FunRich 3.1.3 (Fonseka et al., 2021) software by querying the Gene Ontology database. The Benjamini–Hochberg adjusted p-value (FDR) and fold enrichment cutoffs were 0.001 and 3, respectively. The biological process over-representation analysis was performed using the ClueGO 2.5.7 app (Bindea et al., 2009) of the Cytoscape platform (FDR < 0.05).

### 2.5 Animals

Adult male Wistar rats (280–320 g) were obtained from the Central Animal House at the University of Campinas (CEMIB-UNICAMP; São Paulo, Brazil). All experimental protocols were approved by the Ethics Committee for Animal Use of the UNICAMP (CEUA; Protocol No. 5746-1/2021; 5831-1/2021), following the Brazilian Guidelines for the Production, Maintenance, and Use of Animals for Teaching or Research from the National Council of Control in Animal Experimentation (CONCEA), and the ARRIVE guidelines (Percie du Sert et al., 2020). Three individuals were housed in each cage placed on ventilated shelters at a humidity of 55% ± 5% and a temperature of 24°C ± 1°C under a 12-h light–dark cycle. Animals received filtered water and standard rodent food *ad libitum*.

### 2.6 Langendorff’s isolated perfused heart preparation and measurements of heart contractile function

Heparin (1,000 IU/kg) was previously injected intraperitoneally into the animals to prevent blood clotting, and euthanasia was performed by administering isoflurane overdose, as previously described (Britto-Júnior et al., 2023a). Exsanguination was performed to confirm the euthanasia. The chest was opened, the heart was rapidly excised, the ascending aorta was cannulated, and the heart was mounted on a nonrecirculating Langendorff apparatus. The isolated heart was perfused with Krebs–Henseleit’s solution (pH 7.4, 37°C) equilibrated with a carbogen gas mixture (95% O_2_: 5% CO_2_) at a constant flow (10 mL/min), and left ventricular end-diastolic pressure (LVEDP) was maintained between 4 and 8 mmHg during the initial equilibrium of the experiment (Britto-Júnior et al., 2023a). A water-filled latex balloon, connected to the pressure transducer (MLT1199 BP Transducer, ADInstruments, Inc., Dunedin, NZ), was inserted into the left ventricle (LV) via the mitral valve. Left ventricular systolic pressure (LVSP), left ventricular end-diastolic pressure (LVeDP), and heart rate (HR) were continuously recorded using a PowerLab System (ADInstruments, Inc., Dunedin, NZ). Only hearts that presented a basal heart rate between 250 and 300 bpm were employed in the experiments.

The hearts were allowed to equilibrate for at least 10 min, and the effects of a 1-min infusion (100 μL/min) 6-nitrodopamine (0.01, 0.1, or 1 pM final concentration) were evaluated. Each heart was subjected to only one infusion. Changes were monitored for 30 min. To investigate the synergism between 6-nitrodopamine and noradrenaline in the Langendorff’s perfused heart analysis, the following protocols were employed. One-minute (100 μL/min) infusion of 6-nitrodopamine (0.001 or 0.01 pM, final concentration) was performed, and then, a single bolus of noradrenaline (1 pmol) was administered, and the heart was monitored for 15 min. One heart was used for a single drug and infusion. Data obtained from the Landendorff preparations (heart rate, LVDP, dP/dt max, and RPP) were expressed as left ventricular developed pressure (LVDP), calculated using the following formula: LVSP − LVeDP, and expressed in mmHg. The rate pressure product (RPP) was defined as the product of HR and LVDP: RPP = (HR × LVDP). The maximal rate of increase in the left ventricular pressure (+dP/dt_max_) was monitored continuously using a pressure transducer connected to a PowerLab system (AD Instrument, Australia).

### 2.7 Statistical analysis

Data obtained from the Langendorff preparations were represented by mean ± standard error of the mean (SEM). Comparison between baseline values and those obtained during drug stimulation in the same sample was performed using the paired *t*-test. Comparison between two groups was performed using the unpaired *t*-test. Comparisons among three or more groups were evaluated using one-way analysis of variance (ANOVA), followed by the Newman–Keuls test. *P* < 0.05 was considered statistically significant.

### 2.8 Chemicals and reagents

Noradrenaline was obtained from Cayman Chemicals (Michigan, United States). 6-Nitrodopamine was acquired from Toronto Research Chemicals (Ontario, CA). PureCube Carboxy Agarose Beads were purchased from Cube Biotech (Monheim, Germany). Trypsin**,** the Mem-PER™ Plus Membrane Protein Extraction Kit, Pierce 660 nm Protein Assay, GelCode™ Blue Safe Protein Stain, Orbitrap Exploris 240 (Mass Spectrometer), and Vanquish Neo nanoUPLC System were acquired from Thermo Fisher Scientific (Waltham, MA, United States). 4-NO2-propranolol was synthesized as described elsewhere (Sparaco et al., 2022). Sodium chloride (NaCl), potassium chloride (KCl), calcium chloride (CaCl_2_), magnesium sulfate (MgSO_4_), sodium bicarbonate (NaHCO_3_), potassium phosphate mono-basic (KH_2_PO_4_), and glucose were acquired from Merck KGaA (Darmstadt, Germany).

## 3 Results

### 3.1 Identification of 6-ND targets in cardiomyocytes

The proteins identified from the pre-clearing (PC lane) were discarded from the protein list obtained in the chemical pulldown (PD lane). The remaining proteins were filtered against the proteins listed in the Contaminant Repository for Affinity Purification (CRAPome) 2.0 (Mellacheruvu et al., 2013). At the end of this procedure, 869 potential 6-ND direct and indirect interactors were obtained (Supplementary Table S1). Another pulldown experiment was performed in the presence of an excess of 4-nitro-propranolol, the 6-ND antagonist. Eighty-two proteins were retained on the beads and represent the specific interactors of 6-ND (Table 1), and 52 have already been identified in the pulldown with 6-ND. By subtracting these 52 proteins from the 869 potential 6-ND direct and indirect interactors, a list of 817 proteins was obtained (Supplementary Table S2), which represent the interactors shared between 6-ND and 4-nitropropranolol experiments.

**TABLE 1:** List of proteins retained on the beads following the chemical pulldown experiment performed using an excess of 4-nitro-propranolol.

Uniprot ID	Protein name	Gene name	Sequence coverage [%]	Razor + unique peptides	Unique peptides
P35914	Hydroxymethylglutaryl-CoA lyase, mitochondrial	HMGCL	10.2	4	4
Q96DB5	Regulator of microtubule dynamics protein 1	RMDN1	10.8	4	4
Q9H3N1	Thioredoxin-related transmembrane protein 1	TMX1	12.1	4	4
P07858	Cathepsin B	CTSB	12.4	5	5
P48047	ATP synthase subunit O, mitochondrial	ATP5PO	15	4	4
Q16698	2,4-dienoyl-CoA reductase, mitochondrial	DECR1	15.8	4	4
Q8NBJ7	Inactive C-alpha-formylglycine-generating enzyme 2	SUMF2	16.3	4	4
Q9BPW8	Protein NipSnap homolog 1	NIPSNAP1	16.5	5	4
P51572	B-cell receptor-associated protein 31	BCAP31	17.5	4	4
Q9BVK6	Transmembrane emp24 domain-containing protein 9	TMED9	19.1	5	4
O15400	Syntaxin-7	STX7	19.9	4	4
O95571	Persulfide dioxygenase ETHE1, mitochondrial	ETHE1	20.9	4	4
Q96CN7	Isochorismatase domain-containing protein 1	ISOC1	21.1	4	4
P09429	High-mobility group protein B1	HMGB1	21.4	4	4
P09012	U1 small-nuclear ribonucleoprotein A	SNRPA	22.3	4	3
P62258	14-3-3 protein epsilon	YWHAE	23.1	4	4
P61019	Ras-related protein R2A	RAB2A	23.1	4	2
P30041	Peroxiredoxin-6	PRDX6	23.2	4	4
P61106	Ras-related protein R14	RAB14	23.3	5	5
Q9NXA8	NAD-dependent protein deacylase sirtuin-5, mitochondrial	SIRT5	23.9	6	6
P20340	Ras-related protein R6A	RAB6A	24	6	6
Q9NP72	Ras-related protein R18	RAB18	24.3	4	4
P62826	GTP-binding nuclear protein Ran	RAN	24.5	5	5
Q7Z4W1	L-xylulose reductase	DCXR	24.6	4	4
O95292	Vesicle-associated membrane protein-associated protein B/C	VAPB	24.7	5	4
O15173	Membrane-associated progesterone receptor component 2	PGRMC2	26.5	5	5
Q86V81	THO complex subunit 4	ALYREF	26.8	4	4
P0DPI2	Glutamine amidotransferase-like class 1 domain-containing protein 3A, mitochondrial	GATD3A	27.2	6	6
Q9H9Z2	Protein lin-28 homolog A	LIN28A	27.8	4	4
P27144	Adenylate kinase 4, mitochondrial	AK4	28.7	4	4
P30086	Phosphatidylethanolamine-binding protein 1	BP1	28.9	4	4
P51148	Ras-related protein R5C	RAB5C	29.2	5	4
P61586	Transforming protein RhoA	RHOA	29.5	7	6
Q15691	Microtubule-associated protein RP/EB family member 1	MAPRE1	30.6	5	5
Q9UIJ7	GTP:AMP phosphotransferase AK3, mitochondrial	AK3	33.5	7	7
P63104	14-3-3 protein zeta/delta	YWHAZ	35.5	8	7
P09211	Glutathione S-transferase P	GSTP1	36.2	5	5
O00264	Membrane-associated progesterone receptor component 1	PGRMC1	36.9	8	7
O75947	ATP synthase subunit d, mitochondrial	ATP5PD	37.9	6	6
P46782	40S ribosomal protein S5	RPS5	41.7	8	8
Q99497	Protein/nucleic acid deglycase DJ-1	PARK7	43.9	4	4
Q99714	3-hydroxyacyl-CoA dehydrogenase type-2	HSD17B10	47.9	7	7
P63244	Receptor of activated protein C kinase 1	RACK1	54.9	15	15
O15382	Branched-chain-amino-acid aminotransferase, mitochondrial	BCAT2	12.2	4	4
P23526	Adenylhomocysteinase	AHCY	12.7	5	5
Q8NBX0	Saccharopine dehydrogenase-like oxidoreductase	SCCPDH	13.1	4	4
P09486	SPARC	SPARC	13.2	4	4
P24752	Acetyl-CoA acetyltransferase, mitochondrial	ACAT1	14.3	4	4
Q7L592	Protein arginine methyltransferase NDUFAF7, mitochondrial	NDUFAF7	14.7	4	4
Q13510	Acid ceramidase	ASAH1	14.7	5	5
P82650	28S ribosomal protein S22, mitochondrial	MRPS22	14.7	4	4
P50148	Guanine nucleotide-binding protein G(q) subunit alpha	GNAQ	15.3	4	4
P78310	Coxsackievirus and adenovirus receptor	CXADR	15.3	5	5
P17612	cAMP-dependent protein kinase catalytic subunit alpha	PRKACA	15.7	4	4
P17174	Aspartate aminotransferase, cytoplasmic	GOT1	16.7	4	4
Q15738	Sterol-4-alpha-carboxylate 3-dehydrogenase, decarboxylating	NSDHL	17.2	4	4
Q12907	Vesicular integral-membrane protein VIP36	LMAN2	17.4	6	6
O43464	Serine protease HTRA2, mitochondrial	HTRA2	18.3	5	5
Q9BWD1	Acetyl-CoA acetyltransferase, cytosolic	ACAT2	19.4	4	4
Q9Y371	Endophilin-B1	SH3GLB1	19.5	5	5
Q9H2U2	Inorganic pyrophosphatase 2, mitochondrial	PPA2	20.1	5	5
Q9Y2S7	Polymerase delta-interacting protein 2	POLDIP2	20.4	5	5
Q6NVY1	3-hydroxyisobutyryl-CoA hydrolase, mitochondrial	HIBCH	22	6	6
P52907	F-actin-capping protein subunit alpha-1	CAPZA1	22	4	3
Q9Y3F4	Serine–threonine kinase receptor-associated protein	STRAP	22.6	4	4
A8MXV4	Nucleoside diphosphate-linked moiety X motif 19	NUDT19	23.2	5	5
Q4G0N4	NAD kinase 2, mitochondrial	NADK2	23.5	6	6
Q9BTV4	Transmembrane protein 43	TMEM43	23.5	5	5
P16422	Epithelial cell adhesion molecule	EPCAM	23.6	7	7
Q9BXW7	Haloacid dehalogenase-like hydrolase domain-containing 5	HDHD5	24.1	7	7
O75521	Enoyl-CoA delta isomerase 2, mitochondrial	ECI2	24.9	5	5
P16219	Short-chain-specific acyl-CoA dehydrogenase, mitochondrial	ACADS	25.5	7	7
O43148	mRNA cap guanine-N7 methyltransferase	RNMT	25.8	8	8
O94905	Erlin-2	ERLIN2	26.8	8	8
P00387	NADH-cytochrome b5 reductase 3	CYB5R3	27.6	5	5
P45954	Short-/branched-chain-specific acyl-CoA dehydrogenase, mitochondrial	ACADSB	29.9	8	8
P50453	Serpin B9	SERPINB9	30.6	9	9
P62140	Serine/threonine–protein phosphatase PP1-beta catalytic subunit	PPP1CB	36.4	12	2
O00330	Pyruvate dehydrogenase protein X component, mitochondrial	PDHX	13	5	5
Q9ULV4	Coronin-1C	CORO1C	13.5	6	6
Q9NZW5	MAGUK p55 subfamily member 6	MPP6	16.7	8	8
P04040	Catalase	CAT	21.1	8	8

### 3.2 Functional enrichment analysis of 6-ND targets

**FIGURE 4:**
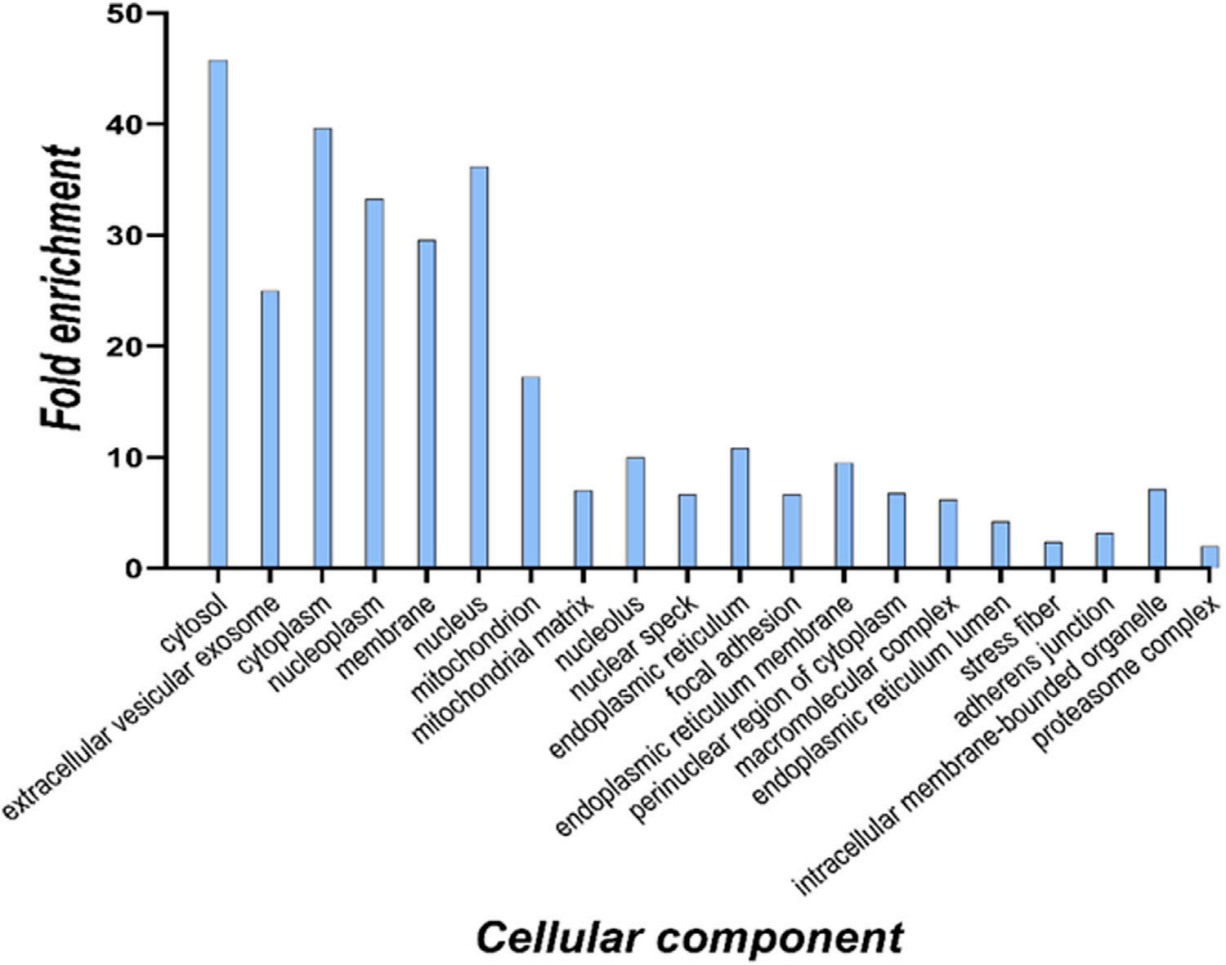
Cellular component analysis of all identified 6-ND targets based on the Gene Ontology database. For each enriched term, the histogram displays the fold enrichment value.

To define cell components enriched in the targets of 6-ND within cardiomyocytes, an over-representation analysis was performed on the 817 proteins in common with the competitor. The identified proteins were functionally analyzed using the FunRich 3.1.3 search engine, in order to interrogate the Gene Ontology database for subcellular localizations. The terms were considered significant when the FDR was <0.001 and the fold enrichment was higher than 3. The results are reported in the histogram in Figure 4.

The cellular component analysis suggested a distribution of potential 6-ND targets through different cell compartments. This finding was expected since the membrane protein extract was partially contaminated also with cytosolic proteins, as suggested by the Western blot assay (Figure 1).

Therefore, in light of our interest in potential cell surface targets, the UniProt “Retrieve/ID Mapping” function (Pundir et al., 2016) was used; the total protein list was further screened according to the Gene Ontology cellular component category, and only those genes whose annotation contained the term “membrane” were considered for further analyses. From the initial list of 869 entries, only 124 (Table 2) were selected because they satisfied the previous restriction criteria.

**TABLE 2:** List of proteins screened according to the Gene Ontology cellular localization in membrane. The UniProt ID, the protein and gene names, the sequence coverage 9(%), and the number of identified peptides are also reported.

Uniprot ID	Protein name	Gene name	Sequence coverage [%]	Razor + unique peptides	Unique peptides
A0FGR8	Extended synaptotagmin-2	ESYT2	12.3	10	10
O00186	Syntaxin-binding protein 3	STXBP3	14.4	10	10
O14672	Disintegrin and metalloproteinase domain-containing protein 10	ADAM10	19.9	15	15
O14936	Peripheral plasma membrane protein CASK	CASK	28.3	25	25
O15031	Plexin-B2	PLXNB2	15.6	23	22
O43278	Kunitz-type protease inhibitor 1	SPINT1	34.8	18	18
Q01518	Adenylyl cyclase-associated protein 1	CAP1	39.6	18	18
O43865	S-adenosylhomocysteine hydrolase-like protein 1	AHCYL1	20.2	12	5
O60488	Long-chain-fatty-acid--CoA ligase 4	ACSL4	13.9	6	6
P10644	cAMP-dependent protein kinase type I-alpha regulatory subunit	PRKAR1A	10	5	5
O75923	Dysferlin	DYSF	12.8	20	20
Q9UKS6	Protein kinase C and casein kinase substrate in neuron protein 3	PACSIN3	11.8	5	5
O75976	Carboxypeptidase D	CPD	21.4	28	28
O94875	Sorbin and SH3 domain-containing protein 2	SORBS2	15.2	12	11
O94973	AP-2 complex subunit alpha-2	AP2A2	42.2	19	19
Q15019	Septin-2	SEPTIN2	37.4	11	11
P78310	Coxsackievirus and adenovirus receptor	CXADR	24.7	10	10
P05067	Amyloid-beta precursor protein	APP	19.5	14	12
P05186	Alkaline phosphatase, tissue-nonspecific isozyme	ALPL	13	5	5
P07384	Calpain-1 catalytic subunit	CAPN1	14.6	10	10
P07947	Tyrosine–protein kinase Yes	YES1	32.2	9	8
P07948	Tyrosine–protein kinase Lyn	LYN	22.1	9	9
P08069	Insulin-like growth factor 1 receptor	IGF1R	11.2	15	12
P15151	Poliovirus receptor	PVR	14.6	4	4
Q9BRK5	45 kDa calcium-binding protein	SDF4	11.6	5	5
P08582	Melanotransferrin	MELTF	16.9	11	11
O75955	Flotillin-1	FLOT1	10.8	5	5
Q6NZI2	Caveolae-associated protein 1	CAVIN1	14.4	5	5
P12830	Cadherin-1	CDH1	11.3	8	7
P12931	Proto-oncogene tyrosine–protein kinase Src	SRC	34.1	16	11
P14735	Insulin-degrading enzyme	IDE	11.7	12	12
P48426	Phosphatidylinositol 5–phosphate 4-kinase type-2 alpha	PIP4K2A	16	6	4
P17655	Calpain-2 catalytic subunit	CAPN2	11.3	7	7
P18084	Integrin beta-5	ITGB5	17.1	12	12
P18564	Integrin beta-6	ITGB6	15.2	10	10
Q13449	Limbic system-associated membrane protein	LSAMP	14.2	5	5
P26232	Catenin alpha-2	CTNNA2	35.4	15	15
P01876	Immunoglobulin heavy constant alpha 1	IGHA1	22.4	7	4
P29323	Ephrin type-B receptor 2	EPHB2	12.6	9	8
P49841	Glycogen synthase kinase-3 beta	GSK3B	17.4	6	4
P31327	Carbamoyl–phosphate synthase [ammonia], mitochondrial	CPS1	23.2	29	29
Q96CX2	BTB/POZ domain-containing protein KCTD12	KCTD12	29.5	11	11
P35241	Radixin	RDX	48	20	20
P35611	Alpha-adducin	ADD1	30.4	19	18
Q9ULV4	Coronin-1C	CORO1C	53.2	25	24
P46940	Ras GTPase-activating-like protein IQGAP1	IQGAP1	12.4	21	21
P31323	cAMP-dependent protein kinase type II-beta regulatory subunit	PRKAR2B	32.3	9	9
P49757	Protein numb homolog	NUMB	14.9	8	8
Q9Y5X3	Sorting nexin-5	SNX5	11.9	4	4
P50570	Dynamin-2	DNM2	10.5	8	8
P53041	Serine/threonine–protein phosphatase 5	PPP5C	12.6	6	6
P53618	Coatomer subunit beta	COPB1	24.8	20	20
P54578	Ubiquitin carboxyl–terminal hydrolase 14	USP14	31.6	14	14
P54753	Ephrin type-B receptor 3	EPHB3	16.2	14	11
Q9H4A6	Golgi phosphoprotein 3	GOLPH3	16.1	4	4
P21796	Voltage-dependent anion-selective channel protein 1	VDAC1	45.6	11	11
P27105	Erythrocyte band 7 integral membrane protein	STOM	46.5	11	11
P08174	Complement decay-accelerating factor	CD55	10.5	5	5
P40123	Adenylyl cyclase-associated protein 2	CAP2	17.6	8	8
Q01650	Large neutral amino acid transporter small subunit 1	SLC7A5	13.6	7	7
Q01844	RNA-binding protein EWS	EWSR1	14.3	7	7
Q02487	Desmocollin-2	DSC2	11.5	9	9
Q06787	Synaptic functional regulator FMR1	FMR1	21.5	13	12
Q08209	Serine/threonine–protein phosphatase 2B catalytic subunit alpha isoform	PPP3CA	18.8	9	6
Q08554	Desmocollin-1	DSC1	10.5	9	9
Q12959	Disks large homolog 1	DLG1	17.3	17	17
Q13153	Serine/threonine–protein kinase PAK 1	PAK1	25.5	5	3
Q13177	Serine/threonine–protein kinase PAK 2	PAK2	27.3	11	5
Q13356	RING-type E3 ubiquitin–protein ligase PPIL2	PPIL2	12.3	5	5
O95210	Starch-binding domain-containing protein 1	STBD1	17.6	5	5
Q13564	NEDD8-activating enzyme E1 regulatory subunit	NAE1	15.7	8	8
Q13586	Stromal interaction molecule 1	STIM1	12.8	7	7
Q13740	CD166 antigen	ALCAM	25.9	14	14
Q13884	Beta-1-syntrophin	SNTB1	17.1	8	8
Q14126	Desmoglein-2	DSG2	18.5	15	15
P09104	Gamma-enolase	ENO2	23.5	5	4
Q14699	Raftlin	RFTN1	33.4	15	15
Q14BN4	Sarcolemmal membrane-associated protein	SLMAP	15.5	11	11
O75781	Paralemmin-1	PALM	42.1	16	14
Q15642	Cdc42-interacting protein 4	TRIP10	33.6	15	15
Q16625	Occludin	OCLN	10.2	5	5
Q5T0N5	Formin-binding protein 1-like	FNBP1L	12.4	8	8
Q5T2T1	MAGUK p55 subfamily member 7	MPP7	11.5	6	6
Q9Y639	Neuroplastin	NPTN	26.4	11	11
Q86X29	Lipolysis-stimulated lipoprotein receptor	LSR	11.7	6	6
Q8IZL8	Proline-, glutamic acid-, and leucine-rich protein 1	PELP1	16.6	13	13
Q8N3R9	MAGUK p55 subfamily member 5	MPP5	25.8	15	15
Q92692	Nectin-2	NECTIN2	23.8	10	10
Q93052	Lipoma-preferred partner	LPP	30.6	17	17
P98172	Ephrin-B1	EFNB1	23.7	5	5
Q96D71	RalBP1-associated Eps domain-containing protein 1	REPS1	11.8	8	8
Q96IF1	LIM domain-containing protein ajuba	AJUBA	16.7	7	7
Q96J84	Kin of IRRE-like protein 1	KIRREL1	10.6	7	7
O43493	Trans-Golgi network integral membrane protein 2	TGOLN2	30.5	13	13
Q99523	Sortilin	SORT1	19	17	17
Q99829	Copine-1	CPNE1	12.8	8	8
Q96QA5	Gasdermin-A	GSDMA	18.4	7	7
P50148	Guanine nucleotide-binding protein G(q) subunit alpha	GNAQ	30.6	10	7
Q9BZF1	Oxysterol-binding protein-related protein 8	OSBPL8	13.3	12	12
Q9H223	EH domain-containing protein 4	EHD4	16.6	7	6
P07858	Cathepsin B	CTSB	19.8	6	6
Q9H4M9	EH domain-containing protein 1	EHD1	23.6	11	7
P16422	Epithelial cell adhesion molecule	EPCAM	33.1	10	10
Q9UBC2	Epidermal growth factor receptor substrate 15-like 1	EPS15L1	16	12	12
Q9UDY2	Tight junction protein ZO-2	TJP2	10.3	9	9
Q9UEY8	Gamma-adducin	ADD3	12.3	8	8
Q9UH65	Switch-associated protein 70	SWAP70	13.8	8	8
Q9UHB6	LIM domain and actin-binding protein 1	LIMA1	24.5	17	17
Q9BRK3	Matrix remodeling-associated protein 8	MXRA8	14.5	8	8
Q9UMX0	Ubiquilin-1	UBQLN1	31.1	10	5
Q9UNF0	Protein kinase C and casein kinase substrate in neuron protein 2	PACSIN2	33.7	16	15
Q9UPT5	Exocyst complex component 7	EXOC7	15.6	11	11
Q14254	Flotillin-2	FLOT2	23.4	10	10
P08138	Tumor necrosis factor receptor superfamily member 16	NGFR	11.9	4	4
P63244	Receptor of activated protein C kinase 1	RACK1	64.7	18	18
Q9Y6I3	Epsin-1	EPN1	13.7	4	4
P35232	Prohibitin	PHB	43.4	10	10
P51148	Ras-related protein Rab-5C	RAB5C	32.9	5	5
P61586	Transforming protein RhoA	RHOA	47.2	8	8
P62491	Ras-related protein Rab-11A	RAB11A	31.5	7	6
P63000	Ras-related C3 botulinum toxin substrate 1	RAC1	38	7	7
P80723	Brain acid-soluble protein 1	BASP1	75.3	9	9
Q9P0L0	Vesicle-associated membrane protein-associated protein A	VAPA	22.9	5	4
Q9Y696	Chloride intracellular channel protein 4	CLIC4	30	4	4

Surprisingly, 116 proteins among the 124 are present in the list of the competitor binding proteins (Table 3). This finding highlights an almost complete overlapping between the sets of membrane protein targets recognized by the two active drugs, suggesting their involvement in stimulation/regulation of common regulative processes.

**TABLE 3:** List of proteins screened according to the Gene Ontology cellular localization in the membrane. The UniProt ID, the protein and gene names, the sequence coverage (%), and the number of identified peptides are also reported. The proteins identified also in the experiment with the competitor are highlighted in bold.

Uniprot ID	Protein name	Gene name	Sequence coverage [%]	Razor + unique peptides	Unique peptides
**A0FGR8**	**Extended synaptotagmin-2**	**ESYT2**	**12.3**	**10**	**10**
**O00186**	**Syntaxin-binding protein 3**	**STXBP3**	**14.4**	**10**	**10**
**O14672**	**Disintegrin and metalloproteinase domain-containing protein 10**	**ADAM10**	**19.9**	**15**	**15**
**O14936**	**Peripheral plasma membrane protein CASK**	**CASK**	**28.3**	**25**	**25**
**O15031**	**Plexin-B2**	**PLXNB2**	**15.6**	**23**	**22**
**O43278**	**Kunitz-type protease inhibitor 1**	**SPINT1**	**34.8**	**18**	**18**
**Q01518**	**Adenylyl cyclase-associated protein 1**	**CAP1**	**39.6**	**18**	**18**
**O43865**	**S-adenosylhomocysteine hydrolase-like protein 1**	**AHCYL1**	**20.2**	**12**	**5**
**O60488**	**Long-chain-fatty-acid--CoA ligase 4**	**ACSL4**	**13.9**	**6**	**6**
**P10644**	**cAMP-dependent protein kinase type I-alpha regulatory subunit**	**PRKAR1A**	**10**	**5**	**5**
**O75923**	**Dysferlin**	**DYSF**	**12.8**	**20**	**20**
**Q9UKS6**	**Protein kinase C and casein kinase substrate in neuron protein 3**	**PACSIN3**	**11.8**	**5**	**5**
**O75976**	**Carboxypeptidase D**	**CPD**	**21.4**	**28**	**28**
**O94875**	**Sorbin and SH3 domain-containing protein 2**	**SORBS2**	**15.2**	**12**	**11**
**O94973**	**AP-2 complex subunit alpha-2**	**AP2A2**	**42.2**	**19**	**19**
**Q15019**	**Septin-2**	**SEPTIN2**	**37.4**	**11**	**11**
**P05067**	**Amyloid-beta precursor protein**	**APP**	**19.5**	**14**	**12**
**P05186**	**Alkaline phosphatase, tissue-nonspecific isozyme**	**ALPL**	**13**	**5**	**5**
**P07384**	**Calpain-1 catalytic subunit**	**CAPN1**	**14.6**	**10**	**10**
**P07947**	**Tyrosine–protein kinase Yes**	**YES1**	**32.2**	**9**	**8**
**P07948**	**Tyrosine–protein kinase Lyn**	**LYN**	**22.1**	**9**	**9**
**P08069**	**Insulin-like growth factor 1 receptor**	**IGF1R**	**11.2**	**15**	**12**
**P15151**	**Poliovirus receptor**	**PVR**	**14.6**	**4**	**4**
**Q9BRK5**	**45-kDa calcium-binding protein**	**SDF4**	**11.6**	**5**	**5**
**P08582**	**Melanotransferrin**	**MELTF**	**16.9**	**11**	**11**
**O75955**	**Flotillin-1**	**FLOT1**	**10.8**	**5**	**5**
**Q6NZI2**	**Caveolae-associated protein 1**	**CAVIN1**	**14.4**	**5**	**5**
**P12830**	**Cadherin-1**	**CDH1**	**11.3**	**8**	**7**
**P12931**	**Proto-oncogene tyrosine–protein kinase Src**	**SRC**	**34.1**	**16**	**11**
**P14735**	**Insulin-degrading enzyme**	**IDE**	**11.7**	**12**	**12**
**P48426**	**Phosphatidylinositol 5-phosphate 4-kinase type-2 alpha**	**PIP4K2A**	**16**	**6**	**4**
**P17655**	**Calpain-2 catalytic subunit**	**CAPN2**	**11.3**	**7**	**7**
**P18084**	**Integrin beta-5**	**ITGB5**	**17.1**	**12**	**12**
**P18564**	**Integrin beta-6**	**ITGB6**	**15.2**	**10**	**10**
**Q13449**	**Limbic system-associated membrane protein**	**LSAMP**	**14.2**	**5**	**5**
**P26232**	**Catenin alpha-2**	**CTNNA2**	**35.4**	**15**	**15**
**P01876**	**Immunoglobulin heavy constant alpha 1**	**IGHA1**	**22.4**	**7**	**4**
**P29323**	**Ephrin type-B receptor 2**	**EPHB2**	**12.6**	**9**	**8**
**P49841**	**Glycogen synthase kinase-3 beta**	**GSK3B**	**17.4**	**6**	**4**
**P31327**	**Carbamoyl–phosphate synthase (ammonia), mitochondrial**	**CPS1**	**23.2**	**29**	**29**
**Q96CX2**	**BTB/POZ domain-containing protein KCTD12**	**KCTD12**	**29.5**	**11**	**11**
**P35241**	**Radixin**	**RDX**	**48**	**20**	**20**
**P35611**	**Alpha-adducin**	**ADD1**	**30.4**	**19**	**18**
**P46940**	**Ras GTPase-activating-like protein IQGAP1**	**IQGAP1**	**12.4**	**21**	**21**
**P31323**	**cAMP-dependent protein kinase type II-beta regulatory subunit**	**PRKAR2B**	**32.3**	**9**	**9**
**P49757**	**Protein numb homolog**	**NUMB**	**14.9**	**8**	**8**
**Q9Y5X3**	**Sorting nexin-5**	**SNX5**	**11.9**	**4**	**4**
**P50570**	**Dynamin-2**	**DNM2**	**10.5**	**8**	**8**
**P53041**	**Serine/threonine–protein phosphatase 5**	**PPP5C**	**12.6**	**6**	**6**
**P53618**	**Coatomer subunit beta**	**COPB1**	**24.8**	**20**	**20**
**P54578**	**Ubiquitin carboxyl-terminal hydrolase 14**	**USP14**	**31.6**	**14**	**14**
**P54753**	**Ephrin type-B receptor 3**	**EPHB3**	**16.2**	**14**	**11**
**Q9H4A6**	**Golgi phosphoprotein 3**	**GOLPH3**	**16.1**	**4**	**4**
**P21796**	**Voltage-dependent anion-selective channel protein 1**	**VDAC1**	**45.6**	**11**	**11**
**P27105**	**Erythrocyte band 7 integral membrane protein**	**STOM**	**46.5**	**11**	**11**
**P08174**	**Complement decay-accelerating factor**	**CD55**	**10.5**	**5**	**5**
**P40123**	**Adenylyl cyclase-associated protein 2**	**CAP2**	**17.6**	**8**	**8**
**Q01650**	**Large neutral amino acid transporter small subunit 1**	**SLC7A5**	**13.6**	**7**	**7**
**Q01844**	**RNA-binding protein EWS**	**EWSR1**	**14.3**	**7**	**7**
**Q02487**	**Desmocollin-2**	**DSC2**	**11.5**	**9**	**9**
**Q06787**	**Synaptic functional regulator FMR1**	**FMR1**	**21.5**	**13**	**12**
**Q08209**	**Serine/threonine–protein phosphatase 2B catalytic subunit alpha isoform**	**PPP3CA**	**18.8**	**9**	**6**
**Q08554**	**Desmocollin-1**	**DSC1**	**10.5**	**9**	**9**
**Q12959**	**Disks large homolog 1**	**DLG1**	**17.3**	**17**	**17**
**Q13153**	**Serine/threonine–protein kinase PAK 1**	**PAK1**	**25.5**	**5**	**3**
**Q13177**	**Serine/threonine–protein kinase PAK 2**	**PAK2**	**27.3**	**11**	**5**
**Q13356**	**RING-type E3 ubiquitin-protein ligase PPIL2**	**PPIL2**	**12.3**	**5**	**5**
**O95210**	**Starch-binding domain-containing protein 1**	**STBD1**	**17.6**	**5**	**5**
**Q13564**	**NEDD8-activating enzyme E1 regulatory subunit**	**NAE1**	**15.7**	**8**	**8**
**Q13586**	**Stromal interaction molecule 1**	**STIM1**	**12.8**	**7**	**7**
**Q13740**	**CD166 antigen**	**ALCAM**	**25.9**	**14**	**14**
**Q13884**	**Beta-1-syntrophin**	**SNTB1**	**17.1**	**8**	**8**
**Q14126**	**Desmoglein-2**	**DSG2**	**18.5**	**15**	**15**
**P09104**	**Gamma-enolase**	**ENO2**	**23.5**	**5**	**4**
**Q14699**	**Raftlin**	**RFTN1**	**33.4**	**15**	**15**
**Q14BN4**	**Sarcolemmal membrane-associated protein**	**SLMAP**	**15.5**	**11**	**11**
**O75781**	**Paralemmin-1**	**PALM**	**42.1**	**16**	**14**
**Q15642**	**Cdc42-interacting protein 4**	**TRIP10**	**33.6**	**15**	**15**
**Q16625**	**Occludin**	**OCLN**	**10.2**	**5**	**5**
**Q5T0N5**	**Formin-binding protein 1-like**	**FNBP1L**	**12.4**	**8**	**8**
**Q5T2T1**	**MAGUK p55 subfamily member 7**	**MPP7**	**11.5**	**6**	**6**
**Q9Y639**	**Neuroplastin**	**NPTN**	**26.4**	**11**	**11**
**Q86X29**	**Lipolysis-stimulated lipoprotein receptor**	**LSR**	**11.7**	**6**	**6**
**Q8IZL8**	**Proline-, glutamic acid-, and leucine-rich protein 1**	**PELP1**	**16.6**	**13**	**13**
**Q8N3R9**	**MAGUK p55 subfamily member 5**	**PALS1**	**25.8**	**15**	**15**
**Q92692**	**Nectin-2**	**NECTIN2**	**23.8**	**10**	**10**
**Q93052**	**Lipoma-preferred partner**	**LPP**	**30.6**	**17**	**17**
**P98172**	**Ephrin-B1**	**EFNB1**	**23.7**	**5**	**5**
**Q96D71**	**RalBP1-associated Eps domain-containing protein 1**	**REPS1**	**11.8**	**8**	**8**
**Q96IF1**	**LIM domain-containing protein ajuba**	**AJUBA**	**16.7**	**7**	**7**
**Q96J84**	**Kin of IRRE-like protein 1**	**KIRREL1**	**10.6**	**7**	**7**
**O43493**	**Trans-Golgi network integral membrane protein 2**	**TGOLN2**	**30.5**	**13**	**13**
**Q99523**	**Sortilin**	**SORT1**	**19**	**17**	**17**
**Q99829**	**Copine-1**	**CPNE1**	**12.8**	**8**	**8**
**Q96QA5**	**Gasdermin-A**	**GSDMA**	**18.4**	**7**	**7**
**Q9BZF1**	**Oxysterol-binding protein-related protein 8**	**OSBPL8**	**13.3**	**12**	**12**
**Q9H223**	**EH domain-containing protein 4**	**EHD4**	**16.6**	**7**	**6**
**Q9H4M9**	**EH domain-containing protein 1**	**EHD1**	**23.6**	**11**	**7**
**Q9UBC2**	**Epidermal growth factor receptor substrate 15-like 1**	**EPS15L1**	**16**	**12**	**12**
**Q9UDY2**	**Tight junction protein ZO-2**	**TJP2**	**10.3**	**9**	**9**
**Q9UEY8**	**Gamma-adducin**	**ADD3**	**12.3**	**8**	**8**
**Q9UH65**	**Switch-associated protein 70**	**SWAP70**	**13.8**	**8**	**8**
**Q9UHB6**	**LIM domain and actin-binding protein 1**	**LIMA1**	**24.5**	**17**	**17**
**Q9BRK3**	**Matrix remodeling-associated protein 8**	**MXRA8**	**14.5**	**8**	**8**
**Q9UMX0**	**Ubiquilin-1**	**UBQLN1**	**31.1**	**10**	**5**
**Q9UNF0**	**Protein kinase C and casein kinase substrate in neuron protein 2**	**PACSIN2**	**33.7**	**16**	**15**
**Q9UPT5**	**Exocyst complex component 7**	**EXOC7**	**15.6**	**11**	**11**
**Q14254**	**Flotillin-2**	**FLOT2**	**23.4**	**10**	**10**
**P08138**	**Tumor necrosis factor receptor superfamily member 16**	**NGFR**	**11.9**	**4**	**4**
**Q9Y6I3**	**Epsin-1**	**EPN1**	**13.7**	**4**	**4**
**P35232**	**Prohibitin**	**PHB**	**43.4**	**10**	**10**
**P62491**	**Ras-related protein Rab-11A**	**RAB11A**	**31.5**	**7**	**6**
**P63000**	**Ras-related C3 botulinum toxin substrate 1**	**RAC1**	**38**	**7**	**7**
**P80723**	**Brain acid-soluble protein 1**	**BASP1**	**75.3**	**9**	**9**
**Q9P0L0**	**Vesicle-associated membrane protein-associated protein A**	**VAPA**	**22.9**	**5**	**4**
**Q9Y696**	**Chloride intracellular channel protein 4**	**CLIC4**	**30**	**4**	**4**

### 3.3 Functional clustering analysis

**FIGURE 5:**
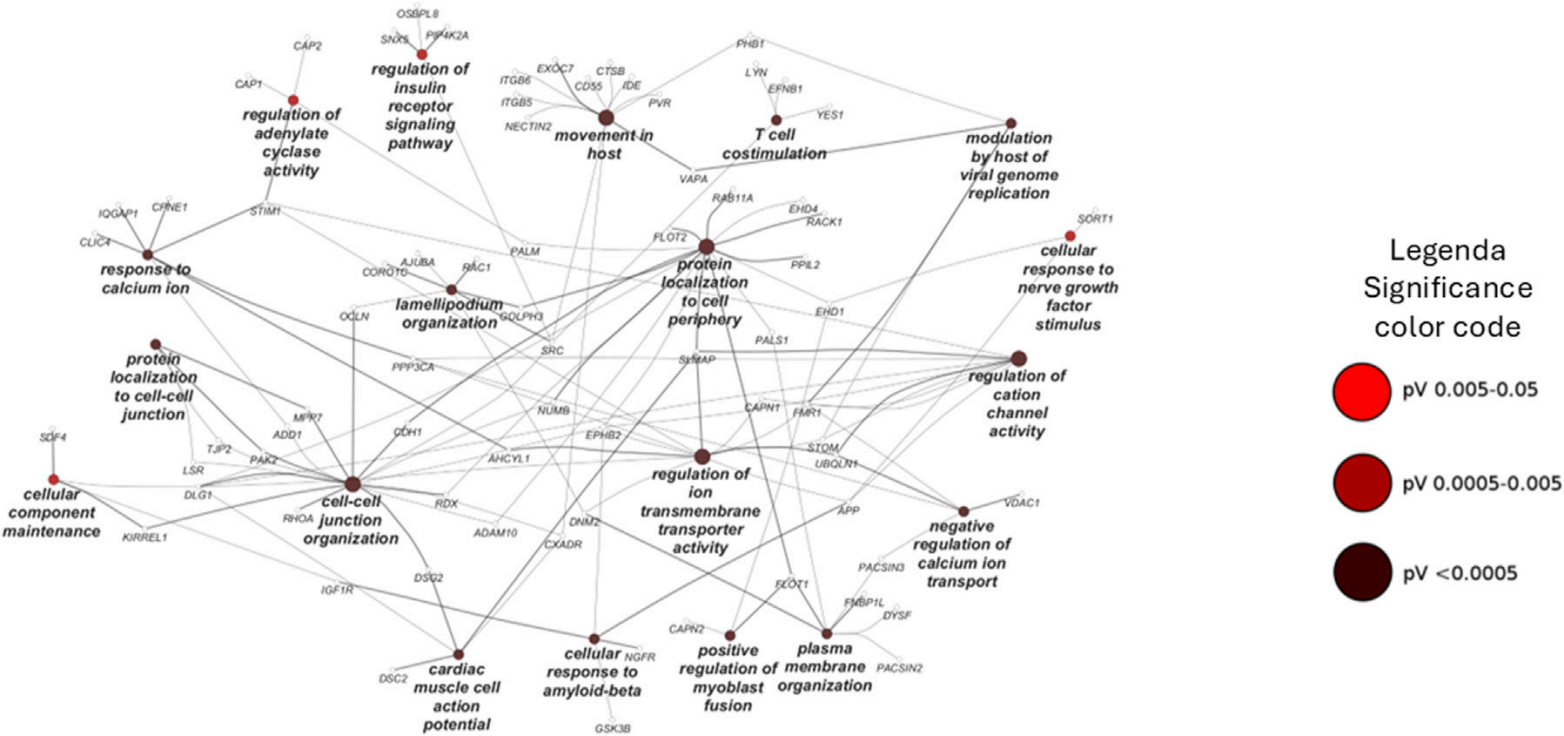
Pathways enrichment analysis through ClueGO based on the biological process database. Network representation of the enriched pathway in the chemical pulldown experiment. The node sizes are proportional to the FDR values.

Cell compartment enrichment analysis was carried out using Funrich 3.1.3 software by querying the Gene Ontology database. The Benjamini–Hochberg-adjusted p-value (FDR) and fold enrichment cutoffs were 0.001 and 3, respectively. The biological process over-representation analysis was performed using the ClueGO 2.5.7 app of the Cytoscape platform (FDR < 0.05), and the functional clustering analysis is represented in Figure 5.

### 3.4 Positive chronotropic and inotropic effects induced by 6-ND in Langendorff’s preparation

**FIGURE 6:**
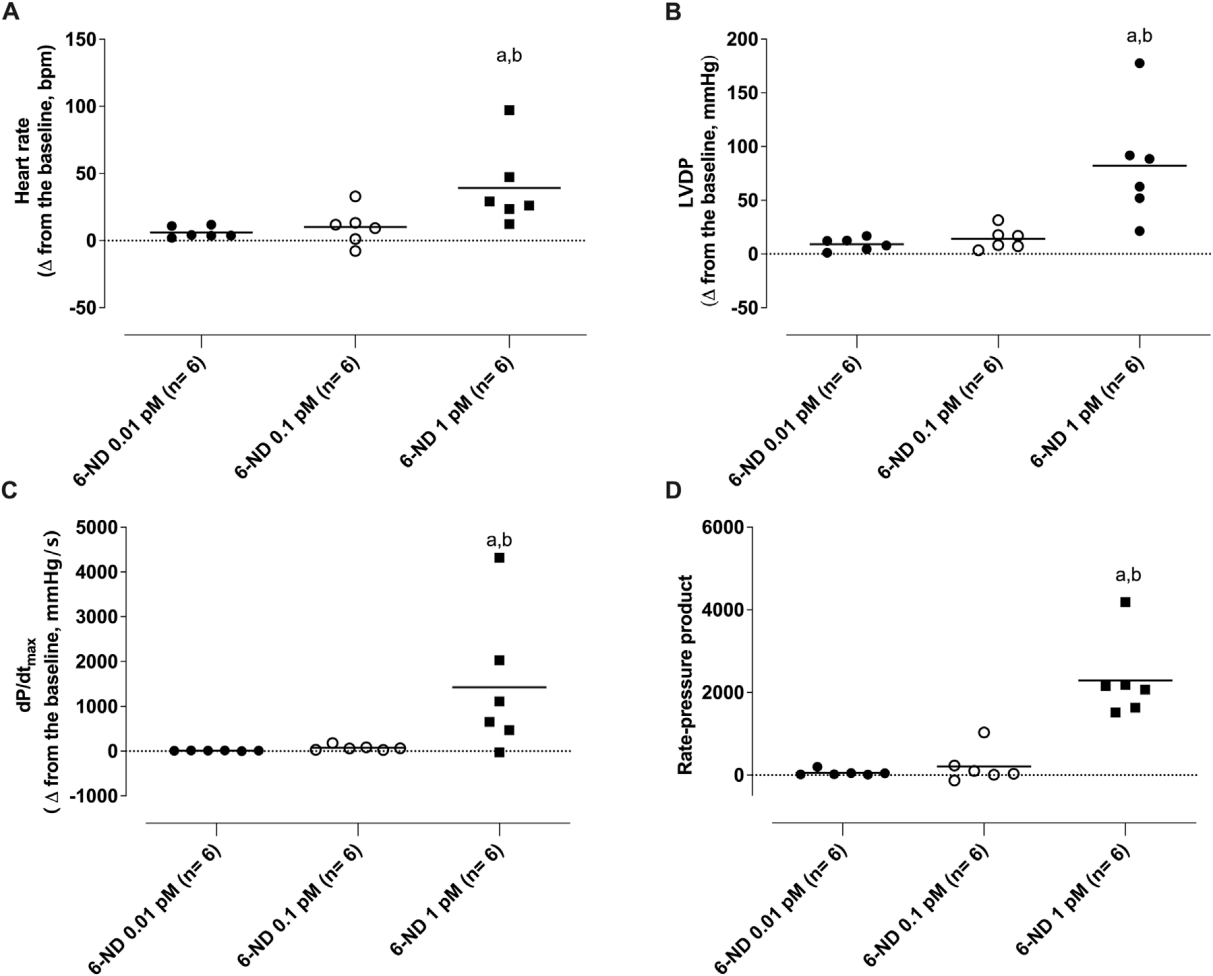
Effect of 1-min infusion of 6-nitrodopamine (6-ND) in the heart rate (HR, **(A)**), left ventricular developed pressure (LVDP, **(B)**), maximum rate of pressure development (dP/dt_max_, **(C)**), and rate pressure product (RPP, **(D)**). ^a^P < 0.05 compared with the lowest concentration of 6-ND (0.01 pM) in each panel; ^b^P < 0.05 compared with the second concentration of 6-ND (0.1 pM) in each panel. NA, noradrenaline.

At low concentrations, 6-nitrodopamine (0.01 and 0.1 pM) had no chronotropic and/or inotropic effect. However, at a higher concentration (1 pM), 6-ND infusion significantly increased the heart rate (Figure 6A), LVDP (Figure 6B), dP/dt (max) (Figure 6C), and RPP (Figure 6D).

### 3.5 Interactions of 6-nitrodopamine with noradrenaline on the isolated rat heart (Langendorff’s preparation)

**FIGURE 7:**
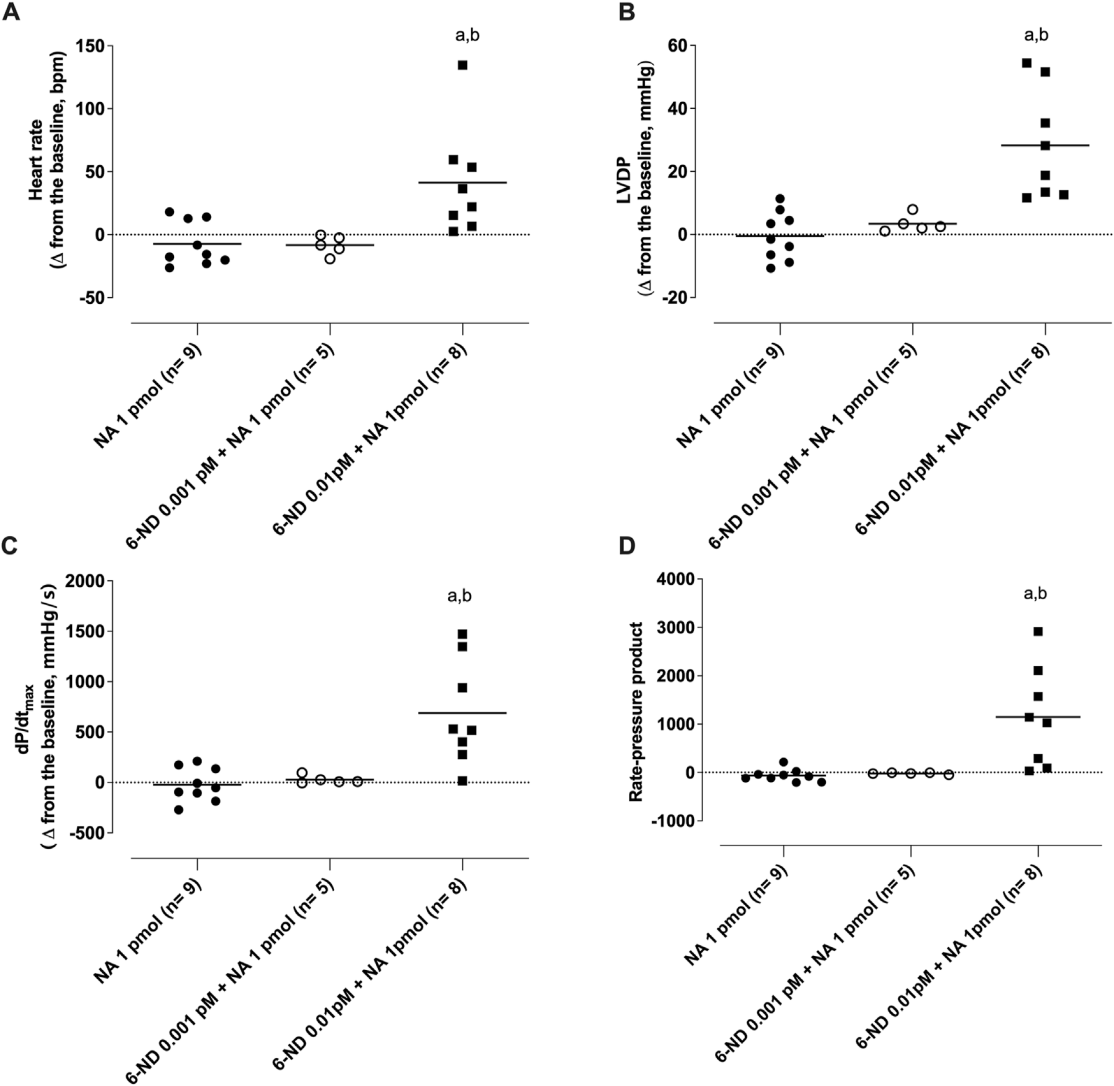
Interaction of 6-nitrodopamine (6-ND) with noradrenaline (NA) on the heart rate (**(A)**), left ventricular developed pressure (LVDP, **(B)**), maximum rate of pressure development (dP/dt_max_, **(C)**), and rate pressure product (RPP, **(D)**). One-minute infusion (100 mL/min) of 6-ND (0.001 or 0.01 pM; right panels) was performed in the absence and presence of a single bolus of noradrenaline (1 pmol). ANOVA, followed by the Newman–Keuls post-test, was applied. ^a^P < 0.05 compared with the NA 1 pmol in each panel; ^b^P < 0.05 compared with the 6-ND 0.001 pM + NA 1 pmol.

Bolus injection of noradrenaline (1 pmol) had no effect on the heart rate frequency (Figure 7A), LVDP (Figure 7B), dPdt (max) (Figure 7C), and RPP (Figure 7D). One-min infusion of 6-nitrodopamine (0.001 pM) alone did not alter any of these parameters either. However, infusion of 6-nitrodopamine (0.01 pM) significantly increased the heart rate frequency (Figure 7A), LVDP (Figure 7B), dP/dt (max) (Figure 7C), and RPP (Figure 7D) when noradrenaline (1 pmol) was injected at the end of the infusion (1 min).

## 4 Discussion

Mammalian hearts express β1- and β2-adrenoceptor subtypes, both of which are involved in the increases in tissue cAMP due to AC activation (Brodde, 2007). Catecholamines such as noradrenaline and adrenaline bind to G-protein–β adrenoceptors, releasing the stimulatory G-protein subunit (Gas) inside the cardiomyocyte, activating AC (Motiejunaite et al., 2021). The generated cAMP binds to protein kinase A (PKA)–R subunits, leading to PKA activation (Liu et al., 2022). Increased PKA activity increases Ca^2+^ levels, leading to enhanced cardiac muscle contractility. Our study using the Langendorff’s preparation clearly demonstrated that 1-min infusion of the perfused heart with a very low concentration of 6-ND (0.1 pM) markedly potentiated the positive chronotropic and inotropic responses induced by noradrenaline. Thus, these pharmacological data seem to be of great value in identifying the 6-ND receptor. Because cAMP-activated PKA is a central regulator of heart chronotropism and inotropism, it is possible that remarkable potentiation caused by 6-ND could be due to AC pathway activation.

The 116 proteins were identified as potential receptors for this novel endogenous catecholamine by using a chemical proteomics approach based on the affinity purification procedure coupled to mass spectrometry for protein identification. The experiments were performed to purify and identify the cardiomyocyte membrane proteins, obtained from cell lysates, bound to 6-ND agarose and inhibited by the selective 6-ND antagonist 4-nitropropranolol (Sparaco et al., 2022; Oliveira et al., 2024a). The number is not surprising since the ligand concentration (in the micromolar or millimolar range) is high to facilitate protein uptake. The AP-MS-based methods present many advantages; they are not time-consuming, do not require the use of specific tools such as antibodies, and allows direct identification of proteins without any bias or prediction. However, it entails limitations since it is an *in vitro* method, and therefore, it can lead to identification of physically real but not physiologically meaningful interactions (Ziegler et al., 2013). Furthermore, false-positive interacting proteins can be extracted upon binding with matrix or with the linker between the beads and the molecule, which can also partially affect the binding process (Tabana et al., 2023). Nevertheless, this approach remains one of the most widely used preliminary and unbiased exploration strategies for exploring protein–ligand interactions and characterizing protein binders to small molecules in various contexts (Babak et al., 2015; Smith et al., 2023; Saltzman et al., 2024). A similar affinity purification method was used to identify dopamine targets in a human embryo kidney cell line (HEK293; Weigert Muñoz et al., 2023). A comparison of the 205 interactors identified in this study with the 869 interactors here reported revealed 30 common proteins. It is worth mentioning that CAP-1, CAP-2, and STIM-1 were absent in the dopamine interactomes.

In the rat isolated right atrium, 6-ND, as a positive chronotropic agent, is 100 times more potent than noradrenaline and adrenaline and 10,000 times more potent than dopamine (Britto-Júnior et al., 2022). As a positive inotropic agent in the rat isolated heart, 6-ND was 1,000 times more potent than noradrenaline and 10,000 times more potent than adrenaline (Britto-Júnior et al., 2022). Thus, the results of the functional pharmacological approaches are of paramount importance to identify the 6-ND receptor.

One distinct characteristic of 6-ND action in the heart, as compared to the classical catecholamines, is its remarkable ability to potentiate the positive chronotropic (Britto-Júnior et al., 2022) and inotropic (Britto-Júnior et al., 2023a) effects induced by noradrenaline, adrenaline, and dopamine and, as shown here, the positive inotropic effect induced by noradrenaline. As mentioned above, binding to transmembrane β-adrenoceptors, to stimulate cAMP-dependent PKA activation in cardiomyocytes, is considered the initial step in cardiomyocyte activation by the classical catecholamines (Liu et al., 2022). It is unlikely that 6-ND acts as a partial agonist on β-adrenoceptors since the increases in the atrial rate induced by PDE-3 inhibitors such as dipyridamole, cilostazol, and milrinone are virtually abolished by pre-incubation with 6-ND, whereas those induced by dopamine, noradrenaline, and adrenaline are unaffected (Britto-Júnior et al., 2023a). Another finding that indicates a lack of effect on β-adrenoceptors is that the pre-incubation of the atria with the protein kinase inhibitor H-89 abolished the increases in the heart rate induced by dopamine, noradrenaline, and adrenaline but only attenuated the increase induced by 6-ND (Britto-Júnior et al., 2023a). The absence of adrenoceptor proteins bound to the 6-ND agarose under our experimental conditions is notable. Thus, modulation of cAMP levels could be a mechanism by which 6-ND could synergize with the classical catecholamines.

Cyclic nucleotide phosphodiesterases (PDEs) modulate cyclic nucleotide signaling by degrading cAMP and 3′,5′-cyclic guanosine monophosphate (cGMP). There are 11 PDE superfamilies [PDE1–11 (Bender and Beavo, 2006)], and the heart/myocytes express mRNA for all but PDE6 (Hashimoto et al., 2018). The major PDE expressed by human cardiomyocytes is PDE1C (Bork et al., 2023), which is a dual PDE substrate, metabolizing both cAMP and cGMP. Inhibition of PDE3 and PDE4 activity increases the atrial rate (Dolce et al., 2021), and PDE1 inhibition enhances cardiomyocyte contractility through a PKA-dependent mechanism (Muller et al., 2021). Therefore, considering that 6-ND does not increase cAMP levels in human platelets (Nash et al., 2022) and of the 817 cardiomyocyte proteins that are bound to 6-ND, none were identified as cAMP- or cGMP-PDE signaling, advocating that 6-ND does not directly modulate PDE activity. It is interesting that the three membrane proteins directly involved in the modulation of AC, namely, cyclase associated protein-1 (CAP-1; Kakurina et al., 2018), CAP-2 (Pelucchi et al., 2023), and STIM-1 (El Assar et al., 2022), are bound to 6-ND agarose, with the binding selectively blocked by 4-nitropropranol, being candidates for the 6-ND receptor.

Purification of the adenylyl cyclase complex from the yeasts *Saccharomyces cerevisiae* (Field et al., 1990) and *Schizosaccharomyces pombe* (Kawamukai et al., 1992) identified the presence of a 70-kDA protein (CAP), whose amino-terminal domain is associated with the AC catalytic area. This protein interaction with AC allows the enzyme to respond appropriately to its regulatory proteins (Vojtek and Cooper, 1993). Homologs for CAP1 and CAP2 have been identified in *Homo sapiens* (Matviw et al., 1992; Yu et al., 1994) and in *Rattus norvegicus* (Swiston et al., 1995); however, the patterns of CAP1 and CAP2 expression varied significantly in adult rat tissues. Interestingly, mRNA levels for CAP2 were significantly higher than those for CAP1 in the rat heart (Swiston et al., 1995).

CAP-1 binds and activates adenylyl cyclase in mammalian cells (Zhang et al., 2021). In PCCL3 thyroid follicular cells, overexpression of CAP-1 caused a marked leftward shift in the forskolin dose–response curve, whereas negative modulation of CAP-1 resulted in a significant rightward shift (Zhang et al., 2021). It is interesting that although 6-ND potentiates the positive chronotropic effect of classical catecholamines, it strongly reduces the positive chronotropic (Kaumann et al., 2009) and inotropic (Christ et al., 2009) effects induced by PDE3 inhibitors, indicating a dual ability to modulate AC. Ablation of CAP2 in mice causes dilated cardiomyopathy associated with severe reduction in the heart rate (Peche et al., 2013). It is interesting that the two human CAP proteins are distinct enough to suggest that they may have different regulatory roles (Yu et al., 1994). Whether they have different ability to modulate AC in the heart is under current investigation. Direct quantification of cAMP levels following stimulation with 6-ND and classical catecholamines may yield additional insights into 6-ND signaling within cardiomyocytes. Furthermore, silencing CAP-1 and CAP-2 mRNA expressions should elucidate the modulatory functions of these proteins in the mechanism of action of 6-ND.

Stromal interaction protein (STIM1) is expressed in cardiomyocytes (Liu et al., 2023) and has a single transmembrane domain (Hooper et al., 2000); it is located in the sarcoplasmic reticulum and plasma membrane (Soboloff et al., 2012), and it is also associated with adenylyl cyclase activation (Motiani et al., 2018). Changes in cytosolic Ca^2+^ levels are known to either enhance or depress cAMP production through various Ca^2+^-sensitive AC isoforms (Willoughby et al., 2012). Of the nine transmembrane AC isoforms described so far, AC1 and AC8 are the major Ca^2+^-activated isoforms, whereas AC5 and AC6 are subjected to direct inhibition by physiological Ca^2+^ in the cytosol. Cardiac-specific deletion of STIM1 in mice causes a reduction in the heart rate and sinus arrest, together with a potentiation of the autonomic response to cholinergic signaling (Zhang et al., 2016). Although the negative chronotropism is compatible with the lack of the 6-ND effect, there is no evidence that 6-ND potentiates cholinergic effects (Oliveira et al., 2024b). It is interesting that STIM1 can also act as a Ca^2+^ sensor by activating store-operated calcium channels (SOCCs) following sarcoplasmic/endoplasmic reticulum Ca^2+^ store depletion (Zhang et al., 2005). STIM1 can bind Ca^2+^ under resting conditions, but, after store depletion, STIM1 can interact with Orai channels to activate the store-operated Ca^2+^ entry (Rosenberg et al., 2021), raising the possibility that 6-ND may modulate calcium transport. Indeed, one of the identified interactors with 6-ND is dysferlin, a 238-kDa transmembrane protein with multiple Ca^2+^-binding domains, which mediates Ca^2+^-dependent membrane fusion in striated muscle cells. Dysferlin-knockout (KO) mice develop a dilated cardiomyopathy characterized by decreased left ventricular ejection fraction and reduced heart rate (Paulke et al., 2024). Although both cardiac phenotypes are compatible with a reduction in 6-ND action, our findings that potentiation by 6-ND on catecholamine action on rat perfused heart is also observed in smooth muscle (Britto-Júnior et al., 2023a) and that the expression of dysferlin is restricted to the skeletal muscle (Chernova et al., 2022) exclude dysferlin as the primary 6-ND target in the heart. Indeed, the cardiac phenotypes in dysferlin-KO mice only become evident in mice over 32 weeks of age (Paulke et al., 2024), whereas the effects reported here are observed acutely. Calpains are 6-ND interactors; they constitute a conservative family of Ca^2+^-dependent intracellular cysteine proteases commonly expressed in all cells (Goll et al., 2003). Two ubiquitous forms of calpains have been identified (Taneike et al., 2011), namely, m- (CAP1) and m-(CAP2), which are activated by micromolar and millimolar calcium concentrations, respectively. Calpain-1 is the primary isoform expressed in cardiomyocytes (Zhang et al., 2024) and plays a critical role in normal heart function by cleaving several target proteins (Patterson et al., 2011). Both Capn1 and Capn2 are heterodimers presenting an 80-kDa catalytic subunit and a common 28-kDa regulatory subunit (calpain 4). Although cardiac-specific deletion of calpain 4 resulted in decreased protein levels of Capn1 and Capn2, no cardiac phenotypes under baseline conditions were observed, a finding not compatible with the acute effects reported here for 6-ND.

Noradrenaline is rapidly degraded by momoamino oxidases (Manzoor and Hoda, 2020), and inhibition of these enzymes by 6-ND could cause potentiation of the inotropic effect of NA. However, 6-ND caused 20% and 30% of inhibition of MAO-A and MAO-B, respectively, at 1 mM; at 100 nM, no inhibition of either enzyme was observed (Fuguhara et al., 2025)*.* Inhibition of catecholamine uptake by 6-ND could potentiate both the positive chronotropic and inotropic effects induced by classical catecholamines. Transporter-mediated uptake plays a major role in determining both the magnitude and duration of the catecholamine effect (Gasser, 2021). There are two types of monoamine transporters, namely, a high-affinity with low capacity to transport monoamines, such as NET (Engelhartet et al., 2020), a low-affinity with high capacity like organic cation transporters (OCT 1–3, Amphoux et al., 2006; Bacq et al., 2012), and the plasma membrane monoamine transporter (PMAT; Torres et al., 2003; Engel et al., 2004). However, it is unlikely that 6-ND could interact with catecholamine uptake since, in our experimental settings, none of these proteins were bound to the 6-ND-derivatized agarose.

The use of human-induced pluripotent stem cell-derived cardiomyocytes is associated with some constraints, such as limited capacity to evaluate contractility, altered maturation properties, and reduced survivability (Shead et al., 2024). Purification of the 6-ND receptor from rat neonatal ventricular myocytes may provide further indication of the importance of the cyclase-associated proteins CAP-1 and CAP-2.

## 5 Conclusion

6-Nitrodopamine-induced potentiation of the catecholamines’ chronotropic and inotropic effects is due to the modulation of adenylyl cyclase activity, probably via direct interactions with CAP-1 and CAP-2.

## Data Availability

The data presented in the study are deposited in the PRIDE repository, accession number PXD066711.

## Supplementary material

Supplementary Figure S1 SDS-PAGE of proteins eluted from the chemical pulldown (lane PD) and from the pre-clearing (lane PC). The molecular weight markers are also reported.

Supplementary Figure S2 Western blot assays for the verification of the fractionated lysis.

## Glossary

6-ND: 6-Nitrodopamine
AC: Adenylyl cyclase
CAP-1: Cyclase-associated protein 1
CAP-2: Cyclase-associated protein 2
STIM1: Stromal interaction molecule 1
hiPSCs: Human-induced pluripotent stem cells
SDS-PAGE: Sodium dodecyl sulfate polyacrylamide gel electrophoresis
GAPDH: Glyceraldehyde-3-phosphate dehydrogenase
HCOOH: Formic acid
ACN: Acetonitrile
DDA: Data-dependent acquisition
CRAPome: Contaminant Repository for Affinity Purification
FDR: False discovery rate
LVEDP: Left ventricular end-diastolic pressure
LVDP: Left ventricular developed pressure
LVSP: Left ventricular systolic pressure
HR: Heart rate
RPP: Rate pressure product
+dP/dtmax: Maximum rate of pressure development
SEM: Standard error of the mean
ANOVA: Analysis of variance
NaCl: Sodium chloride
KCl: Potassium chloride
**CaCl**_**2**_: Calcium chloride
**MgSO**_**4**_: Magnesium sulfate
**NaHCO**_**3**_: Sodium bicarbonate
**KH**_**2**_ **PO**_**4**_: Potassium phosphate mono-basic
PC lane: Pre-clearing lane
PD lane: Pulldown lane
PKA: Protein kinase A
PDE: Phosphodiesterase
cGMP: Cyclic guanosine monophosphate
UPLC: Ultra performance liquid chromatography
PMAT: Plasma membrane monoamine transporter
NET: Norepinephrine transporter
OCT: Organic cation transporter
SOCCs: Store-operated calcium channels
