# Supplementary figures and images for: The identification of adenylyl cyclase modulators as potential receptors for 6-nitrodopamine in human-induced pluripotent stem cell (hiPSC)-derived cardiomyocytes and their relevance in heart inotropism
Source: Front Pharmacol. 2025 Aug 11;16:1597035. doi: 10.3389/fphar.2025.1597035 (PMC12375930; doi:10.3389/fphar.2025.1597035)

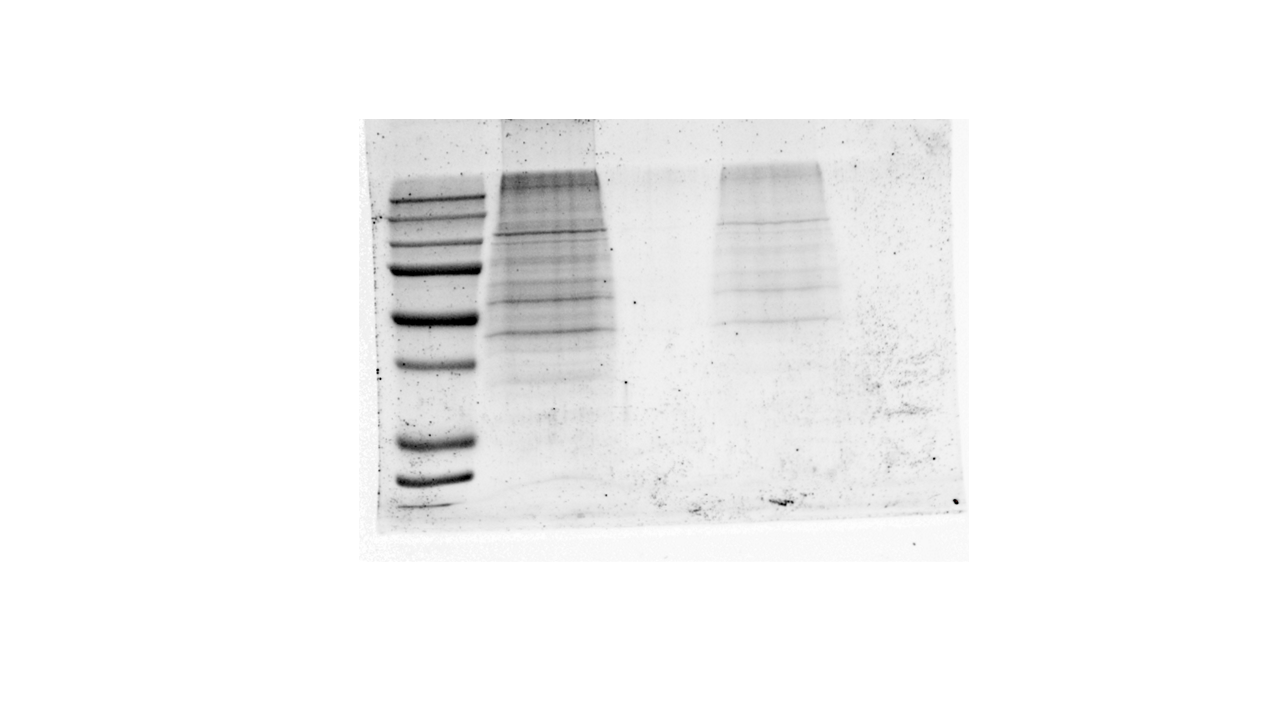

Supplement: Supplementary file 1 [file Image2.tif]

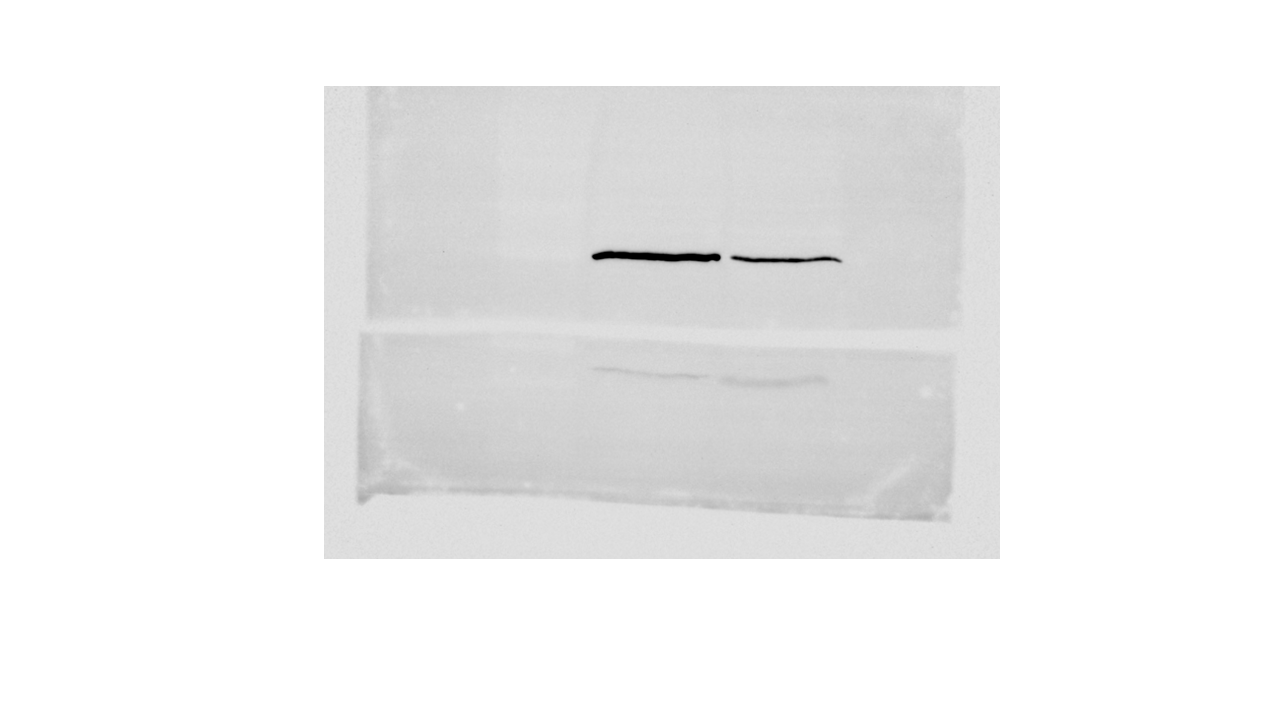

Supplement: Supplementary file 2 [file Image1.tif]
