# Supplementary material for: The identification of adenylyl cyclase modulators as potential receptors for 6-nitrodopamine in human-induced pluripotent stem cell (hiPSC)-derived cardiomyocytes and their relevance in heart inotropism
Source: Front Pharmacol. 2025 Aug 11;16:1597035. doi: 10.3389/fphar.2025.1597035 (PMC12375930; doi:10.3389/fphar.2025.1597035)
Supplement: Supplementary file 3 [file Table1.doc]

**Table S1.** List of proteins filtered from the proteins present in the contaminant Repository for Affinity Purification (CRAPome) 2.0, and representing the potential y-nitrodopamine (6-ND) direct and indirect interactors.

|  | **Uniprot ID** | **Protein name** | **Gene name** | **Sequence coverage [%]** | **Razor + unique peptides** | **Unique peptides** |
| --- | --- | --- | --- | --- | --- | --- |
| 1 | P35556 | Fibrillin-2 | FBN2 | 13.2 | 34 | 34 |
| 2 | O15020 | Spectrin beta chain. non-erythrocytic 2 | SPTBN2 | 14.4 | 20 | 20 |
| 3 | A2RRP1 | Neuroblastoma-amplified sequence | NBAS | 13.2 | 30 | 30 |
| 4 | P12270 | Nucleoprotein TPR | TPR | 34.2 | 76 | 76 |
| 5 | O75165 | DnaJ homolog subfamily C member 13 | DNAJC13 | 14.4 | 26 | 26 |
| 6 | P49750 | YLP motif-containing protein 1 | YLPM1 | 17.2 | 28 | 28 |
| 7 | O75923 | Dysferlin | DYSF | 12.8 | 20 | 20 |
| 8 | P55196 | Afadin | AFDN | 21.2 | 34 | 34 |
| 9 | O15031 | Plexin-B2 | PLXNB2 | 15.6 | 23 | 22 |
| 10 | P07942 | Laminin subunit beta-1 | LAMB1 | 28.2 | 39 | 39 |
| 11 | P52948 | Nuclear pore complex protein Nup98-Nup96 | NUP98 | 10.6 | 20 | 20 |
| 12 | P55268 | Laminin subunit beta-2 | LAMB2 | 22.1 | 32 | 32 |
| 13 | P46940 | Ras GTPase-activating-like protein IQGAP1 | IQGAP1 | 12.4 | 21 | 21 |
| 14 | Q9UBG0 | C-type mannose receptor 2 | MRC2 | 12.1 | 15 | 15 |
| 15 | P31327 | Carbamoyl-phosphate synthase [ammonia]. mitochondrial | CPS1 | 23.2 | 29 | 29 |
| 16 | P08069 | Insulin-like growth factor 1 receptor | IGF1R | 11.2 | 15 | 12 |
| 17 | P49790 | Nuclear pore complex protein Nup153 | NUP153 | 11.2 | 12 | 12 |
| 18 | O75976 | Carboxypeptidase D | CPD | 21.4 | 28 | 28 |
| 19 | Q8WX93 | Palladin | PALLD | 10.7 | 14 | 14 |
| 20 | P41252 | Isoleucine--tRNA ligase. cytoplasmic | IARS | 10.8 | 13 | 13 |
| 21 | Q14896 | Myosin-binding protein C. cardiac-type | MYBPC3 | 12 | 12 | 12 |
| 22 | Q86VP6 | Cullin-associated NEDD8-dissociated protein 1 | CAND1 | 21.4 | 23 | 23 |
| 23 | P55265 | Double-stranded RNA-specific adenosine deaminase | ADAR | 31.6 | 43 | 43 |
| 24 | O75054 | Immunoglobulin superfamily member 3 | IGSF3 | 11.1 | 12 | 12 |
| 25 | Q92896 | Golgi apparatus protein 1 | GLG1 | 16.9 | 18 | 18 |
| 26 | Q9UDY2 | Tight junction protein ZO-2 | TJP2 | 10.3 | 9 | 9 |
| 27 | O94979 | Protein transport protein Sec31A | SEC31A | 17.3 | 19 | 19 |
| 28 | P11498 | Pyruvate carboxylase. mitochondrial | PC | 19.7 | 19 | 19 |
| 29 | Q16531 | DNA damage-binding protein 1 | DDB1 | 16.8 | 17 | 17 |
| 30 | Q92900 | Regulator of nonsense transcripts 1 | UPF1 | 15.1 | 16 | 16 |
| 31 | O94875 | Sorbin and SH3 domain-containing protein 2 | SORBS2 | 15.2 | 12 | 11 |
| 32 | Q9H2P0 | Activity-dependent neuroprotector homeobox protein | ADNP | 16.1 | 14 | 14 |
| 33 | O14980 | Exportin-1 | XPO1 | 26.2 | 25 | 25 |
| 34 | Q14126 | Desmoglein-2 | DSG2 | 18.5 | 15 | 15 |
| 35 | Q8IX01 | SURP and G-patch domain-containing protein 2 | SUGP2 | 14.6 | 16 | 16 |
| 36 | Q8IZL8 | Proline-. glutamic acid- and leucine-rich protein 1 | PELP1 | 16.6 | 13 | 13 |
| 37 | O15042 | U2 snRNP-associated SURP motif-containing protein | U2SURP | 14.5 | 16 | 16 |
| 38 | Q8IXT5 | RNA-binding protein 12B | RBM12B | 12.6 | 7 | 7 |
| 39 | P14735 | Insulin-degrading enzyme | IDE | 11.7 | 12 | 12 |
| 40 | P42285 | Exosome RNA helicase MTR4 | MTREX | 11.7 | 14 | 14 |
| 41 | P29323 | Ephrin type-B receptor 2 | EPHB2 | 12.6 | 9 | 8 |
| 42 | Q5JRX3 | Presequence protease. mitochondrial | PITRM1 | 28.4 | 28 | 28 |
| 43 | Q7L014 | Probable ATP-dependent RNA helicase DDX46 | DDX46 | 17.8 | 18 | 18 |
| 44 | Q9NWH9 | SAFB-like transcription modulator | SLTM | 14.3 | 13 | 13 |
| 45 | Q96KR1 | Zinc finger RNA-binding protein | ZFR | 29.8 | 28 | 28 |
| 46 | O76041 | Nebulette | NEBL | 22.9 | 23 | 23 |
| 47 | O00291 | Huntingtin-interacting protein 1 | HIP1 | 23.9 | 24 | 24 |
| 48 | O14974 | Protein phosphatase 1 regulatory subunit 12A | PPP1R12A | 19.8 | 18 | 18 |
| 49 | Q9H2U1 | ATP-dependent DNA/RNA helicase DHX36 | DHX36 | 11.5 | 10 | 10 |
| 50 | Q14157 | Ubiquitin-associated protein 2-like | UBAP2L | 13.3 | 11 | 11 |
| 51 | Q9ULD0 | 2-oxoglutarate dehydrogenase-like. mitochondrial | OGDHL | 10.3 | 5 | 5 |
| 52 | Q9HCE1 | Helicase MOV-10 | MOV10 | 15.3 | 15 | 15 |
| 53 | Q5T8P6 | RNA-binding protein 26 | RBM26 | 12 | 12 | 12 |
| 54 | P49916 | DNA ligase 3 | LIG3 | 15.5 | 16 | 16 |
| 55 | P23378 | Glycine dehydrogenase (decarboxylating). mitochondrial | GLDC | 15 | 14 | 14 |
| 56 | P55060 | Exportin-2 | CSE1L | 18 | 17 | 17 |
| 57 | P54753 | Ephrin type-B receptor 3 | EPHB3 | 16.2 | 14 | 11 |
| 58 | P33176 | Kinesin-1 heavy chain | KIF5B | 38.2 | 33 | 27 |
| 59 | Q9HC35 | Echinoderm microtubule-associated protein-like 4 | EML4 | 10.8 | 10 | 10 |
| 60 | O75400 | Pre-mRNA-processing factor 40 homolog A | PRPF40A | 13.5 | 12 | 12 |
| 61 | Q9NZ08 | Endoplasmic reticulum aminopeptidase 1 | ERAP1 | 14 | 12 | 12 |
| 62 | Q96T37 | RNA-binding protein 15 | RBM15 | 24.6 | 20 | 20 |
| 63 | P53618 | Coatomer subunit beta | COPB1 | 24.8 | 20 | 20 |
| 64 | P49588 | Alanine--tRNA ligase. cytoplasmic | AARS | 21.5 | 21 | 21 |
| 65 | P57740 | Nuclear pore complex protein Nup107 | NUP107 | 23.2 | 18 | 18 |
| 66 | Q99460 | 26S proteasome non-ATPase regulatory subunit 1 | PSMD1 | 16.5 | 13 | 13 |
| 67 | Q6UB35 | Monofunctional C1-tetrahydrofolate synthase. mitochondrial | MTHFD1L | 27.5 | 26 | 26 |
| 68 | Q9NXF1 | Testis-expressed protein 10 | TEX10 | 11.3 | 9 | 9 |
| 69 | Q99613 | Eukaryotic translation initiation factor 3 subunit C | EIF3C | 28.3 | 30 | 30 |
| 70 | P10253 | Lysosomal alpha-glucosidase | GAA | 14.2 | 11 | 11 |
| 71 | P26232 | Catenin alpha-2 | CTNNA2 | 35.4 | 15 | 15 |
| 72 | O14936 | Peripheral plasma membrane protein CASK | CASK | 28.3 | 25 | 25 |
| 73 | O60462 | Neuropilin-2 | NRP2 | 10.2 | 8 | 8 |
| 74 | O00192 | Armadillo repeat protein deleted in velo-cardio-facial syndrome | ARVCF | 20.5 | 14 | 14 |
| 75 | Q13620 | Cullin-4B | CUL4B | 15.2 | 14 | 9 |
| 76 | O94973 | AP-2 complex subunit alpha-2 | AP2A2 | 42.2 | 19 | 19 |
| 77 | Q8IWX7 | Protein unc-45 homolog B | UNC45B | 28 | 24 | 24 |
| 78 | P98175 | RNA-binding protein 10 | RBM10 | 21.3 | 18 | 16 |
| 79 | P55786 | Puromycin-sensitive aminopeptidase | NPEPPS | 32 | 28 | 28 |
| 80 | P52789 | Hexokinase-2 | HK2 | 35.8 | 30 | 30 |
| 81 | A0FGR8 | Extended synaptotagmin-2 | ESYT2 | 12.3 | 10 | 10 |
| 82 | P78344 | Eukaryotic translation initiation factor 4 gamma 2 | EIF4G2 | 25.8 | 22 | 22 |
| 83 | Q92973 | Transportin-1 | TNPO1 | 17 | 13 | 10 |
| 84 | Q15031 | Probable leucine--tRNA ligase. mitochondrial | LARS2 | 18.6 | 16 | 16 |
| 85 | P11586 | C-1-tetrahydrofolate synthase. cytoplasmic | MTHFD1 | 35.6 | 30 | 30 |
| 86 | Q7Z2W4 | Zinc finger CCCH-type antiviral protein 1 | ZC3HAV1 | 15.9 | 11 | 11 |
| 87 | Q9BXJ9 | N-alpha-acetyltransferase 15. NatA auxiliary subunit | NAA15 | 21.2 | 24 | 18 |
| 88 | Q9BZF1 | Oxysterol-binding protein-related protein 8 | OSBPL8 | 13.3 | 12 | 12 |
| 89 | P56192 | Methionine--tRNA ligase. cytoplasmic | MARS | 13.1 | 11 | 11 |
| 90 | Q9UL12 | Sarcosine dehydrogenase. mitochondrial | SARDH | 16.8 | 15 | 14 |
| 91 | O00462 | Beta-mannosidase | MANBA | 19.5 | 17 | 16 |
| 92 | Q01780 | Exosome component 10 | EXOSC10 | 12.9 | 10 | 10 |
| 93 | P98194 | Calcium-transporting ATPase type 2C member 1 | ATP2C1 | 12.1 | 9 | 9 |
| 94 | Q9BZJ0 | Crooked neck-like protein 1 | CRNKL1 | 12.1 | 10 | 10 |
| 95 | Q12959 | Disks large homolog 1 | DLG1 | 17.3 | 17 | 17 |
| 96 | Q7Z2K6 | Endoplasmic reticulum metallopeptidase 1 | ERMP1 | 15.8 | 12 | 12 |
| 97 | Q13200 | 26S proteasome non-ATPase regulatory subunit 2 | PSMD2 | 19.2 | 14 | 14 |
| 98 | P49756 | RNA-binding protein 25 | RBM25 | 12.2 | 10 | 10 |
| 99 | Q9HCS7 | Pre-mRNA-splicing factor SYF1 | XAB2 | 21.4 | 16 | 16 |
| 100 | Q08554 | Desmocollin-1 | DSC1 | 10.5 | 9 | 9 |
| 101 | Q02487 | Desmocollin-2 | DSC2 | 11.5 | 9 | 9 |
| 102 | Q9UI47 | Catenin alpha-3 | CTNNA3 | 24.9 | 20 | 20 |
| 103 | P55157 | Microsomal triglyceride transfer protein large subunit | MTTP | 20.7 | 18 | 18 |
| 104 | P50570 | Dynamin-2 | DNM2 | 10.5 | 8 | 8 |
| 105 | P29590 | Protein PML | PML | 17.3 | 15 | 15 |
| 106 | P12830 | Cadherin-1 | CDH1 | 11.3 | 8 | 7 |
| 107 | Q99959 | Plakophilin-2 | PKP2 | 30.1 | 28 | 28 |
| 108 | Q9NTZ6 | RNA-binding protein 12 | RBM12 | 11.3 | 9 | 9 |
| 109 | Q9UL18 | Protein argonaute-1 | AGO1 | 14.8 | 6 | 6 |
| 110 | Q9UKV8 | Protein argonaute-2 | AGO2 | 19.6 | 15 | 9 |
| 111 | P06737 | Glycogen phosphorylase. liver form | PYGL | 21.3 | 9 | 9 |
| 112 | P11217 | Glycogen phosphorylase. muscle form | PYGM | 35.5 | 18 | 18 |
| 113 | P11171 | Protein 4.1 | EPB41 | 21.9 | 11 | 11 |
| 114 | Q92598 | Heat shock protein 105 kDa | HSPH1 | 22.8 | 16 | 16 |
| 115 | Q9UBB9 | Tuftelin-interacting protein 11 | TFIP11 | 11.9 | 10 | 10 |
| 116 | Q9GZR7 | ATP-dependent RNA helicase DDX24 | DDX24 | 10.1 | 8 | 8 |
| 117 | P45974 | Ubiquitin carboxyl-terminal hydrolase 5 | USP5 | 13.8 | 9 | 9 |
| 118 | Q9BUQ8 | Probable ATP-dependent RNA helicase DDX23 | DDX23 | 30.2 | 26 | 26 |
| 119 | Q6IN85 | Serine/threonine-protein phosphatase 4 regulatory subunit 3A | PPP4R3A | 10.3 | 6 | 6 |
| 120 | Q14BN4 | Sarcolemmal membrane-associated protein | SLMAP | 15.5 | 11 | 11 |
| 121 | Q9H269 | Vacuolar protein sorting-associated protein 16 homolog | VPS16 | 10.7 | 8 | 8 |
| 122 | Q9UBC2 | Epidermal growth factor receptor substrate 15-like 1 | EPS15L1 | 16 | 12 | 12 |
| 123 | Q8TCJ2 | Dolichyl-diphosphooligosaccharide--protein glycosyltransferase subunit STT3B | STT3B | 15.3 | 17 | 17 |
| 124 | Q2Q1W2 | E3 ubiquitin-protein ligase TRIM71 | TRIM71 | 15.2 | 9 | 9 |
| 125 | Q05682 | Caldesmon | CALD1 | 37.5 | 39 | 39 |
| 126 | P78536 | Disintegrin and metalloproteinase domain-containing protein 17 | ADAM17 | 11.7 | 10 | 10 |
| 127 | O60341 | Lysine-specific histone demethylase 1A | KDM1A | 10.2 | 8 | 8 |
| 128 | Q14566 | DNA replication licensing factor MCM6 | MCM6 | 16.4 | 14 | 14 |
| 129 | P09327 | Villin-1 | VIL1 | 12.9 | 9 | 9 |
| 130 | P55884 | Eukaryotic translation initiation factor 3 subunit B | EIF3B | 15.6 | 14 | 14 |
| 131 | Q99459 | Cell division cycle 5-like protein | CDC5L | 24.9 | 18 | 18 |
| 132 | Q9BQ52 | Zinc phosphodiesterase ELAC protein 2 | ELAC2 | 13.9 | 11 | 11 |
| 133 | P52756 | RNA-binding protein 5 | RBM5 | 19.4 | 12 | 11 |
| 134 | Q99523 | Sortilin | SORT1 | 19 | 17 | 17 |
| 135 | Q09161 | Nuclear cap-binding protein subunit 1 | NCBP1 | 29.2 | 18 | 18 |
| 136 | Q96QK1 | Vacuolar protein sorting-associated protein 35 | VPS35 | 30.4 | 22 | 22 |
| 137 | O43747 | AP-1 complex subunit gamma-1 | AP1G1 | 18.1 | 13 | 13 |
| 138 | P25205 | DNA replication licensing factor MCM3 | MCM3 | 19.2 | 16 | 16 |
| 139 | Q93034 | Cullin-5 | CUL5 | 11.5 | 9 | 9 |
| 140 | O43290 | U4/U6.U5 tri-snRNP-associated protein 1 | SART1 | 27.8 | 24 | 24 |
| 141 | Q96GQ7 | Probable ATP-dependent RNA helicase DDX27 | DDX27 | 12.9 | 10 | 10 |
| 142 | P22735 | Protein-glutamine gamma-glutamyltransferase K | TGM1 | 15.5 | 12 | 12 |
| 143 | Q13616 | Cullin-1 | CUL1 | 10.4 | 9 | 9 |
| 144 | O94874 | E3 UFM1-protein ligase 1 | UFL1 | 23 | 19 | 19 |
| 145 | P22059 | Oxysterol-binding protein 1 | OSBP | 10.9 | 7 | 7 |
| 146 | P46087 | Probable 28S rRNA (cytosine(4447)-C(5))-methyltransferase | NOP2 | 28.1 | 20 | 20 |
| 147 | O75152 | Zinc finger CCCH domain-containing protein 11A | ZC3H11A | 31 | 21 | 21 |
| 148 | Q8WUF5 | RelA-associated inhibitor | PPP1R13L | 15.5 | 10 | 10 |
| 149 | Q9H9E3 | Conserved oligomeric Golgi complex subunit 4 | COG4 | 12.7 | 9 | 9 |
| 150 | Q13618 | Cullin-3 | CUL3 | 19.4 | 13 | 13 |
| 151 | O95479 | GDH/6PGL endoplasmic bifunctional protein | H6PD | 25.2 | 20 | 20 |
| 152 | O75534 | Cold shock domain-containing protein E1 | CSDE1 | 30.6 | 26 | 26 |
| 153 | O43264 | Centromere/kinetochore protein zw10 homolog | ZW10 | 13.7 | 9 | 9 |
| 154 | Q9BWU0 | Kanadaptin | SLC4A1AP | 10.7 | 8 | 8 |
| 155 | Q9UBV2 | Protein sel-1 homolog 1 | SEL1L | 12.5 | 9 | 9 |
| 156 | Q9Y4W6 | AFG3-like protein 2 | AFG3L2 | 16.3 | 14 | 14 |
| 157 | Q9P2I0 | Cleavage and polyadenylation specificity factor subunit 2 | CPSF2 | 14.1 | 12 | 12 |
| 158 | P18084 | Integrin beta-5 | ITGB5 | 17.1 | 12 | 12 |
| 159 | Q9UGP8 | Translocation protein SEC63 homolog | SEC63 | 14.9 | 12 | 12 |
| 160 | Q8TB22 | Spermatogenesis-associated protein 20 | SPATA20 | 18.8 | 13 | 13 |
| 161 | P47897 | Glutamine--tRNA ligase | QARS | 29.7 | 22 | 22 |
| 162 | O14639 | Actin-binding LIM protein 1 | ABLIM1 | 11.7 | 7 | 7 |
| 163 | Q13033 | Striatin-3 | STRN3 | 11.8 | 6 | 6 |
| 164 | Q9Y263 | Phospholipase A-2-activating protein | PLAA | 23.1 | 15 | 15 |
| 165 | Q14694 | Ubiquitin carboxyl-terminal hydrolase 10 | USP10 | 16.5 | 11 | 11 |
| 166 | Q13617 | Cullin-2 | CUL2 | 11.1 | 9 | 9 |
| 167 | P05067 | Amyloid-beta precursor protein | APP | 19.5 | 14 | 12 |
| 168 | Q9Y4K0 | Lysyl oxidase homolog 2 | LOXL2 | 12.3 | 9 | 9 |
| 169 | Q96D71 | RalBP1-associated Eps domain-containing protein 1 | REPS1 | 11.8 | 8 | 8 |
| 170 | Q969S9 | Ribosome-releasing factor 2. mitochondrial | GFM2 | 26.6 | 20 | 20 |
| 171 | Q15437 | Protein transport protein Sec23B | SEC23B | 18.4 | 9 | 9 |
| 172 | Q08J23 | tRNA (cytosine(34)-C(5))-methyltransferase | NSUN2 | 13.3 | 10 | 10 |
| 173 | O95140 | Mitofusin-2 | MFN2 | 20.9 | 13 | 13 |
| 174 | O14964 | Hepatocyte growth factor-regulated tyrosine kinase substrate | HGS | 12.4 | 10 | 10 |
| 175 | O43815 | Striatin | STRN | 17.7 | 12 | 11 |
| 176 | Q8TCS8 | Polyribonucleotide nucleotidyltransferase 1. mitochondrial | PNPT1 | 22.2 | 17 | 17 |
| 177 | P18564 | Integrin beta-6 | ITGB6 | 15.2 | 10 | 10 |
| 178 | Q12797 | Aspartyl/asparaginyl beta-hydroxylase | ASPH | 31.7 | 21 | 21 |
| 179 | O43719 | HIV Tat-specific factor 1 | HTATSF1 | 11.1 | 9 | 9 |
| 180 | Q9NW13 | RNA-binding protein 28 | RBM28 | 30 | 21 | 21 |
| 181 | P51178 | 1-phosphatidylinositol 4.5-bisphosphate phosphodiesterase delta-1 | PLCD1 | 18.4 | 13 | 13 |
| 182 | Q01813 | ATP-dependent 6-phosphofructokinase. platelet type | PFKP | 37 | 27 | 24 |
| 183 | Q9UHB6 | LIM domain and actin-binding protein 1 | LIMA1 | 24.5 | 17 | 17 |
| 184 | P08237 | ATP-dependent 6-phosphofructokinase. muscle type | PFKM | 19.9 | 11 | 10 |
| 185 | Q8IZ83 | Aldehyde dehydrogenase family 16 member A1 | ALDH16A1 | 13.6 | 9 | 9 |
| 186 | P17858 | ATP-dependent 6-phosphofructokinase. liver type | PFKL | 16.5 | 7 | 7 |
| 187 | Q9BZL4 | Protein phosphatase 1 regulatory subunit 12C | PPP1R12C | 21.5 | 12 | 12 |
| 188 | Q96MU7 | YTH domain-containing protein 1 | YTHDC1 | 11.3 | 8 | 8 |
| 189 | Q14684 | Ribosomal RNA processing protein 1 homolog B | RRP1B | 19 | 12 | 12 |
| 190 | O14672 | Disintegrin and metalloproteinase domain-containing protein 10 | ADAM10 | 19.9 | 15 | 15 |
| 191 | P13807 | Glycogen [starch] synthase. muscle | GYS1 | 26.7 | 18 | 18 |
| 192 | Q99567 | Nuclear pore complex protein Nup88 | NUP88 | 16.7 | 9 | 9 |
| 193 | Q96J84 | Kin of IRRE-like protein 1 | KIRREL1 | 10.6 | 7 | 7 |
| 194 | Q96RP9 | Elongation factor G. mitochondrial | GFM1 | 33.4 | 27 | 27 |
| 195 | Q9UPT5 | Exocyst complex component 7 | EXOC7 | 15.6 | 11 | 11 |
| 196 | Q86VS8 | Protein Hook homolog 3 | HOOK3 | 20.8 | 14 | 14 |
| 197 | Q8IYQ7 | Threonine synthase-like 1 | THNSL1 | 20.7 | 13 | 13 |
| 198 | Q9Y6D9 | Mitotic spindle assembly checkpoint protein MAD1 | MAD1L1 | 32.3 | 29 | 29 |
| 199 | Q9Y4W2 | Ribosomal biogenesis protein LAS1L | LAS1L | 18.5 | 12 | 12 |
| 200 | Q8TBA6 | Golgin subfamily A member 5 | GOLGA5 | 26.7 | 16 | 16 |
| 201 | Q6PJT7 | Zinc finger CCCH domain-containing protein 14 | ZC3H14 | 31.7 | 19 | 19 |
| 202 | Q13835 | Plakophilin-1 | PKP1 | 12.6 | 9 | 9 |
| 203 | Q9NY33 | Dipeptidyl peptidase 3 | DPP3 | 18.3 | 11 | 11 |
| 204 | Q9BQ39 | ATP-dependent RNA helicase DDX50 | DDX50 | 24.8 | 18 | 16 |
| 205 | P54802 | Alpha-N-acetylglucosaminidase | NAGLU | 26.2 | 14 | 14 |
| 206 | P07384 | Calpain-1 catalytic subunit | CAPN1 | 14.6 | 10 | 10 |
| 207 | O00461 | Golgi integral membrane protein 4 | GOLIM4 | 29.3 | 17 | 17 |
| 208 | O00429 | Dynamin-1-like protein | DNM1L | 34.1 | 17 | 17 |
| 209 | O00471 | Exocyst complex component 5 | EXOC5 | 12.9 | 9 | 9 |
| 210 | Q9H307 | Pinin | PNN | 18.1 | 16 | 16 |
| 211 | Q9NYY8 | FAST kinase domain-containing protein 2. mitochondrial | FASTKD2 | 14.6 | 9 | 9 |
| 212 | P46199 | Translation initiation factor IF-2. mitochondrial | MTIF2 | 15.5 | 10 | 10 |
| 213 | P33993 | DNA replication licensing factor MCM7 | MCM7 | 27 | 17 | 17 |
| 214 | Q08945 | FACT complex subunit SSRP1 | SSRP1 | 27.2 | 16 | 16 |
| 215 | Q9BW92 | Threonine--tRNA ligase. mitochondrial | TARS2 | 24.8 | 16 | 16 |
| 216 | Q8IVL5 | Prolyl 3-hydroxylase 2 | P3H2 | 27.4 | 20 | 20 |
| 217 | P35611 | Alpha-adducin | ADD1 | 30.4 | 19 | 18 |
| 218 | Q13330 | Metastasis-associated protein MTA1 | MTA1 | 28.3 | 19 | 13 |
| 219 | P48147 | Prolyl endopeptidase | PREP | 11.7 | 7 | 7 |
| 220 | Q96AQ6 | Pre-B-cell leukemia transcription factor-interacting protein 1 | PBXIP1 | 11.1 | 8 | 8 |
| 221 | Q99797 | Mitochondrial intermediate peptidase | MIPEP | 36.2 | 25 | 25 |
| 222 | Q9NRL3 | Striatin-4 | STRN4 | 14.5 | 9 | 7 |
| 223 | P49959 | Double-strand break repair protein MRE11 | MRE11 | 11.9 | 8 | 8 |
| 224 | Q04446 | 1.4-alpha-glucan-branching enzyme | GBE1 | 10.4 | 6 | 6 |
| 225 | Q96RQ3 | Methylcrotonoyl-CoA carboxylase subunit alph mitochondrial | MCCC1 | 28 | 16 | 16 |
| 226 | O95573 | Long-chain-fatty-acid--CoA ligase 3 | ACSL3 | 28.5 | 16 | 14 |
| 227 | P23327 | Sarcoplasmic reticulum histidine-rich calcium-binding protein | HRC | 41.8 | 23 | 23 |
| 228 | P08582 | Melanotransferrin | MELTF | 16.9 | 11 | 11 |
| 229 | P17655 | Calpain-2 catalytic subunit | CAPN2 | 11.3 | 7 | 7 |
| 230 | Q96T51 | RUN and FYVE domain-containing protein 1 | RUFY1 | 14.3 | 9 | 8 |
| 231 | O60488 | Long-chain-fatty-acid--CoA ligase 4 | ACSL4 | 13.9 | 6 | 6 |
| 232 | Q9UEY8 | Gamma-adducin | ADD3 | 12.3 | 8 | 8 |
| 233 | P52888 | Thimet oligopeptidase | THOP1 | 10 | 6 | 6 |
| 234 | Q06210 | Glutamine--fructose-6-phosphate aminotransferase [isomerizing] 1 | GFPT1 | 15.2 | 7 | 7 |
| 235 | Q9H078 | Caseinolytic peptidase B protein homolog | CLPB | 19.2 | 13 | 13 |
| 236 | Q9H078 | Caseinolytic peptidase B protein homolog | CLPB | 19.2 | 13 | 13 |
| 237 | Q14444 | Caprin-1 | CAPRIN1 | 22.4 | 16 | 16 |
| 238 | Q96AC1 | Fermitin family homolog 2 | FERMT2 | 19 | 11 | 11 |
| 239 | Q8ND24 | RING finger protein 214 | RNF214 | 23.3 | 14 | 14 |
| 240 | O15254 | Peroxisomal acyl-coenzyme A oxidase 3 | ACOX3 | 14.6 | 10 | 10 |
| 241 | O43395 | U4/U6 small nuclear ribonucleoprotein Prp3 | PRPF3 | 35.4 | 23 | 23 |
| 242 | Q9UKF6 | Cleavage and polyadenylation specificity factor subunit 3 | CPSF3 | 15.9 | 10 | 10 |
| 243 | Q13948 | Protein CASP | CUX1 | 22.9 | 14 | 14 |
| 244 | Q13586 | Stromal interaction molecule 1 | STIM1 | 12.8 | 7 | 7 |
| 245 | Q8N3R9 | MAGUK p55 subfamily member 5 | PALS1 | 25.8 | 15 | 15 |
| 246 | P23142 | Fibulin-1 | FBLN1 | 20.5 | 15 | 15 |
| 247 | Q6NUQ4 | Transmembrane protein 214 | TMEM214 | 16.1 | 10 | 10 |
| 248 | P49915 | GMP synthase [glutamine-hydrolyzing] | GMPS | 20.9 | 15 | 15 |
| 249 | Q7Z417 | Nuclear fragile X mental retardation-interacting protein 2 | NUFIP2 | 11.5 | 7 | 7 |
| 250 | O96005 | Cleft lip and palate transmembrane protein 1 | CLPTM1 | 14.3 | 9 | 9 |
| 251 | Q9H6T3 | RNA polymerase II-associated protein 3 | RPAP3 | 16.1 | 9 | 9 |
| 252 | Q9P0U4 | CXXC-type zinc finger protein 1 | CXXC1 | 11.9 | 7 | 7 |
| 253 | Q96FV9 | THO complex subunit 1 | THOC1 | 29.5 | 18 | 18 |
| 254 | Q9NVP1 | ATP-dependent RNA helicase DDX18 | DDX18 | 23.7 | 15 | 15 |
| 255 | Q86SF2 | N-acetylgalactosaminyltransferase 7 | GALNT7 | 17.8 | 11 | 11 |
| 256 | P54136 | Arginine--tRNA ligase. cytoplasmic | RARS | 43.6 | 25 | 25 |
| 257 | Q9H089 | Large subunit GTPase 1 homolog | LSG1 | 14.7 | 10 | 10 |
| 258 | P07225 | Vitamin K-dependent protein S | PROS1 | 16.9 | 10 | 10 |
| 259 | O94776 | Metastasis-associated protein MTA2 | MTA2 | 22.5 | 10 | 10 |
| 260 | Q9BW27 | Nuclear pore complex protein Nup85 | NUP85 | 18.6 | 13 | 13 |
| 261 | O43301 | Heat shock 70 kDa protein 12A | HSPA12A | 19.1 | 12 | 12 |
| 262 | Q15061 | WD repeat-containing protein 43 | WDR43 | 11.2 | 7 | 7 |
| 263 | O75746 | Calcium-binding mitochondrial carrier protein Aralar1 | SLC25A12 | 14 | 4 | 4 |
| 264 | Q15582 | Transforming growth factor-beta-induced protein ig-h3 | TGFBI | 17.9 | 10 | 10 |
| 265 | O76094 | Signal recognition particle subunit SRP72 | SRP72 | 33.8 | 25 | 25 |
| 266 | Q9UJS0 | Calcium-binding mitochondrial carrier protein Aralar2 | SLC25A13 | 36.1 | 18 | 13 |
| 267 | P02545 | Prelamin-A/C | LMNA | 20.2 | 12 | 12 |
| 268 | Q9BZE4 | Nucleolar GTP-binding protein 1 | GTPBP4 | 18.6 | 11 | 11 |
| 269 | P08253 | 72 kDa type IV collagenase | MMP2 | 16.5 | 9 | 9 |
| 270 | Q96SI9 | Spermatid perinuclear RNA-binding protein | STRBP | 27.7 | 13 | 13 |
| 271 | Q15397 | Pumilio homolog 3 | PUM3 | 14 | 10 | 10 |
| 272 | Q96BP3 | Peptidylprolyl isomerase domain and WD repeat-containing protein 1 | PPWD1 | 16.7 | 12 | 12 |
| 273 | Q6PI48 | Aspartate--tRNA ligase. mitochondrial | DARS2 | 14.7 | 9 | 9 |
| 274 | Q5K4L6 | Solute carrier family 27 member 3 | SLC27A3 | 15.5 | 8 | 8 |
| 275 | O15231 | Zinc finger protein 185 | ZNF185 | 23.7 | 13 | 13 |
| 276 | O95453 | Poly(A)-specific ribonuclease PARN | PARN | 23.5 | 13 | 13 |
| 277 | Q9NW82 | WD repeat-containing protein 70 | WDR70 | 20.9 | 12 | 12 |
| 278 | Q567U6 | Coiled-coil domain-containing protein 93 | CCDC93 | 23.9 | 15 | 15 |
| 279 | Q8IYK4 | Procollagen galactosyltransferase 2 | COLGALT2 | 10.2 | 5 | 5 |
| 280 | O00116 | Alkyldihydroxyacetonephosphate synthase. peroxisomal | AGPS | 13.7 | 8 | 8 |
| 281 | P42357 | Histidine ammonia-lyase | HAL | 11.9 | 8 | 8 |
| 282 | P35475 | Alpha-L-iduronidase | IDUA | 10.4 | 7 | 7 |
| 283 | Q9H4A4 | Aminopeptidase B | RNPEP | 11.2 | 6 | 6 |
| 284 | Q9ULX6 | A-kinase anchor protein 8-like | AKAP8L | 18.6 | 11 | 11 |
| 285 | Q8NBJ5 | Procollagen galactosyltransferase 1 | COLGALT1 | 17.2 | 12 | 12 |
| 286 | Q9Y5K6 | CD2-associated protein | CD2AP | 14.4 | 7 | 7 |
| 287 | Q86X29 | Lipolysis-stimulated lipoprotein receptor | LSR | 11.7 | 6 | 6 |
| 288 | Q9NVI7 | ATPase family AAA domain-containing protein 3A | ATAD3A | 32.2 | 20 | 7 |
| 289 | Q06787 | Synaptic functional regulator FMR1 | FMR1 | 21.5 | 13 | 12 |
| 290 | P43155 | Carnitine O-acetyltransferase | CRAT | 20 | 11 | 11 |
| 291 | O43252 | Bifunctional 3-phosphoadenosine 5-phosphosulfate synthase 1 | PAPSS1 | 13.6 | 7 | 7 |
| 292 | P13797 | Plastin-3 | PLS3 | 34.1 | 19 | 14 |
| 293 | P49757 | Protein numb homolog | NUMB | 14.9 | 8 | 8 |
| 294 | O60826 | Coiled-coil domain-containing protein 22 | CCDC22 | 13.9 | 8 | 8 |
| 295 | Q53F19 | Nuclear cap-binding protein subunit 3 | NCBP3 | 15.8 | 9 | 9 |
| 296 | Q9UJ14 | Glutathione hydrolase 7 | GGT7 | 13.7 | 8 | 8 |
| 297 | Q7L775 | EPM2A-interacting protein 1 | EPM2AIP1 | 10.2 | 5 | 5 |
| 298 | Q9NVH0 | Exonuclease 3-5 domain-containing protein 2 | EXD2 | 12.4 | 7 | 7 |
| 299 | P13796 | Plastin-2 | LCP1 | 18.3 | 6 | 6 |
| 300 | Q9UBU9 | Nuclear RNA export factor 1 | NXF1 | 24.1 | 12 | 12 |
| 301 | Q5T0N5 | Formin-binding protein 1-like | FNBP1L | 12.4 | 8 | 8 |
| 302 | P00734 | Prothrombin | F2 | 10.3 | 6 | 6 |
| 303 | Q14738 | Serine/threonine-protein phosphatase 2A 56 kDa regulatory subunit delta isoform | PPP2R5D | 10.3 | 5 | 4 |
| 304 | Q03252 | Lamin-B2 | LMNB2 | 14.2 | 6 | 6 |
| 305 | Q9NQW7 | Xaa-Pro aminopeptidase 1 | XPNPEP1 | 16.4 | 8 | 8 |
| 306 | Q9UJV9 | Probable ATP-dependent RNA helicase DDX41 | DDX41 | 18.8 | 10 | 10 |
| 307 | P08240 | Signal recognition particle receptor subunit alpha | SRPRA | 14.9 | 7 | 7 |
| 308 | P18887 | DNA repair protein XRCC1 | XRCC1 | 22.1 | 10 | 10 |
| 309 | P09960 | Leukotriene A-4 hydrolase | LTA4H | 21.4 | 10 | 10 |
| 310 | O76031 | ATP-dependent Clp protease ATP-binding subunit clpX-like. mitochondrial | CLPX | 15.8 | 8 | 8 |
| 311 | P23588 | Eukaryotic translation initiation factor 4B | EIF4B | 35.4 | 22 | 22 |
| 312 | Q9UH65 | Switch-associated protein 70 | SWAP70 | 13.8 | 8 | 8 |
| 313 | Q9H0B6 | Kinesin light chain 2 | KLC2 | 11.1 | 4 | 4 |
| 314 | Q9H845 | Acyl-CoA dehydrogenase family member 9. mitochondrial | ACAD9 | 37.7 | 25 | 25 |
| 315 | P02771 | Alpha-fetoprotein | AFP | 24.8 | 12 | 12 |
| 316 | Q9NSK0 | Kinesin light chain 4 | KLC4 | 13.4 | 5 | 5 |
| 317 | P35241 | Radixin | RDX | 48 | 20 | 20 |
| 318 | O95208 | Epsin-2 | EPN2 | 12.9 | 7 | 7 |
| 319 | Q01844 | RNA-binding protein EWS | EWSR1 | 14.3 | 7 | 7 |
| 320 | Q06124 | Tyrosine-protein phosphatase non-receptor type 11 | PTPN11 | 10.6 | 6 | 6 |
| 321 | Q15642 | Cdc42-interacting protein 4 | TRIP10 | 33.6 | 15 | 15 |
| 322 | Q15637 | Splicing factor 1 | SF1 | 21 | 14 | 14 |
| 323 | Q14677 | Clathrin interactor 1 | CLINT1 | 29.1 | 15 | 15 |
| 324 | P27694 | Replication protein A 70 kDa DNA-binding subunit | RPA1 | 23.4 | 12 | 12 |
| 325 | Q8TAT6 | Nuclear protein localization protein 4 homolog | NPLOC4 | 24.5 | 13 | 13 |
| 326 | O00541 | Pescadillo homolog | PES1 | 16.2 | 10 | 10 |
| 327 | Q6NYC8 | Phostensin | PPP1R18 | 17.5 | 9 | 9 |
| 328 | O00186 | Syntaxin-binding protein 3 | STXBP3 | 14.4 | 10 | 10 |
| 329 | Q96AX1 | Vacuolar protein sorting-associated protein 33A | VPS33A | 19.6 | 11 | 11 |
| 330 | P61764 | Syntaxin-binding protein 1 | STXBP1 | 12.5 | 7 | 7 |
| 331 | Q9BTC8 | Metastasis-associated protein MTA3 | MTA3 | 20 | 6 | 6 |
| 332 | Q6L8Q7 | 2.5-phosphodiesterase 12 | PDE12 | 11.5 | 6 | 6 |
| 333 | P61221 | ATP-binding cassette sub-family E member 1 | ABCE1 | 25.2 | 14 | 14 |
| 334 | Q4G0J3 | La-related protein 7 | LARP7 | 15.3 | 8 | 8 |
| 335 | P17812 | CTP synthase 1 | CTPS1 | 26.7 | 16 | 14 |
| 336 | Q6UN15 | Pre-mRNA 3-end-processing factor FIP1 | FIP1L1 | 22.4 | 12 | 12 |
| 337 | Q9BYC5 | Alpha-(1.6)-fucosyltransferase | FUT8 | 12.9 | 8 | 8 |
| 338 | Q15833 | Syntaxin-binding protein 2 | STXBP2 | 36.8 | 20 | 20 |
| 339 | Q5JTV8 | Torsin-1A-interacting protein 1 | TOR1AIP1 | 12.2 | 5 | 5 |
| 340 | Q8IXH7 | Negative elongation factor C/D | NELFCD | 21.2 | 10 | 10 |
| 341 | Q8NHH9 | Atlastin-2 | ATL2 | 29.8 | 17 | 17 |
| 342 | Q15650 | Activating signal cointegrator 1 | TRIP4 | 13.1 | 7 | 7 |
| 343 | O00567 | Nucleolar protein 56 | NOP56 | 40.1 | 27 | 27 |
| 344 | Q93052 | Lipoma-preferred partner | LPP | 30.6 | 17 | 17 |
| 345 | Q969N2 | GPI transamidase component PIG-T | PIGT | 12.8 | 7 | 7 |
| 346 | Q8WX92 | Negative elongation factor B | NELFB | 18.1 | 11 | 11 |
| 347 | Q9NRF8 | CTP synthase 2 | CTPS2 | 19.5 | 8 | 8 |
| 348 | Q13123 | Protein Red | IK | 45.6 | 23 | 23 |
| 349 | Q9HCD5 | Nuclear receptor coactivator 5 | NCOA5 | 30.2 | 14 | 14 |
| 350 | Q5T2T1 | MAGUK p55 subfamily member 7 | MPP7 | 11.5 | 6 | 6 |
| 351 | Q5T160 | Probable arginine--tRNA ligase. mitochondrial | RARS2 | 19 | 13 | 13 |
| 352 | P08842 | Steryl-sulfatase | STS | 11.3 | 7 | 7 |
| 353 | Q8NHP8 | Putative phospholipase B-like 2 | PLBD2 | 10 | 4 | 4 |
| 354 | Q9UHD8 | Septin-9 | SEPTIN9 | 26.3 | 14 | 14 |
| 355 | Q53GS9 | U4/U6.U5 tri-snRNP-associated protein 2 | USP39 | 26.9 | 13 | 13 |
| 356 | Q8NE62 | Choline dehydrogenase. mitochondrial | CHDH | 12.1 | 7 | 7 |
| 357 | Q08380 | Galectin-3-binding protein | LGALS3BP | 17.8 | 8 | 8 |
| 358 | Q07866 | Kinesin light chain 1 | KLC1 | 24.6 | 13 | 10 |
| 359 | Q8WYA6 | Beta-catenin-like protein 1 | CTNNBL1 | 30.2 | 19 | 19 |
| 360 | Q5VV42 | Threonylcarbamoyladenosine tRNA methylthiotransferase | CDKAL1 | 19.5 | 10 | 10 |
| 361 | Q13740 | CD166 antigen | ALCAM | 25.9 | 14 | 14 |
| 362 | Q9NRW7 | Vacuolar protein sorting-associated protein 45 | VPS45 | 24.9 | 14 | 14 |
| 363 | Q9H4L4 | Sentrin-specific protease 3 | SENP3 | 13.4 | 6 | 6 |
| 364 | Q9BY44 | Eukaryotic translation initiation factor 2A | EIF2A | 26.3 | 12 | 12 |
| 365 | O14545 | TRAF-type zinc finger domain-containing protein 1 | TRAFD1 | 12.5 | 6 | 6 |
| 366 | Q10471 | Polypeptide N-acetylgalactosaminyltransferase 2 | GALNT2 | 36.1 | 18 | 18 |
| 367 | Q14168 | MAGUK p55 subfamily member 2 | MPP2 | 27.4 | 14 | 14 |
| 368 | P49023 | Paxillin | PXN | 17.6 | 9 | 9 |
| 369 | Q86U44 | N6-adenosine-methyltransferase catalytic subunit | METTL3 | 12.8 | 6 | 6 |
| 370 | Q9H0L4 | Cleavage stimulation factor subunit 2 tau variant | CSTF2T | 35.6 | 17 | 10 |
| 371 | Q10472 | Polypeptide N-acetylgalactosaminyltransferase 1 | GALNT1 | 14.5 | 7 | 7 |
| 372 | Q4G176 | Acyl-CoA synthetase family member 3. mitochondrial | ACSF3 | 22.4 | 10 | 10 |
| 373 | Q9P2K5 | Myelin expression factor 2 | MYEF2 | 26 | 15 | 15 |
| 374 | A0AV96 | RNA-binding protein 47 | RBM47 | 12.8 | 5 | 5 |
| 375 | Q13131 | 5-AMP-activated protein kinase catalytic subunit alpha-1 | PRKAA1 | 12.7 | 6 | 6 |
| 376 | O15371 | Eukaryotic translation initiation factor 3 subunit D | EIF3D | 39.1 | 18 | 18 |
| 377 | Q96HC4 | PDZ and LIM domain protein 5 | PDLIM5 | 37.1 | 17 | 17 |
| 378 | Q96KC8 | DnaJ homolog subfamily C member 1 | DNAJC1 | 12.6 | 6 | 6 |
| 379 | Q8IY67 | Ribonucleoprotein PTB-binding 1 | RAVER1 | 34 | 17 | 17 |
| 380 | Q7Z739 | YTH domain-containing family protein 3 | YTHDF3 | 14.5 | 5 | 4 |
| 381 | Q9NRR5 | Ubiquilin-4 | UBQLN4 | 16.5 | 5 | 5 |
| 382 | Q86UE4 | Protein LYRIC | MTDH | 25.8 | 12 | 12 |
| 383 | Q86W50 | RNA N6-adenosine-methyltransferase METTL16 | METTL16 | 10 | 5 | 5 |
| 384 | O95470 | Sphingosine-1-phosphate lyase 1 | SGPL1 | 19.7 | 11 | 11 |
| 385 | Q8N1G4 | Leucine-rich repeat-containing protein 47 | LRRC47 | 39.1 | 23 | 23 |
| 386 | Q9NPI6 | mRNA-decapping enzyme 1A | DCP1A | 22 | 8 | 8 |
| 387 | O95793 | Double-stranded RNA-binding protein Staufen homolog 1 | STAU1 | 18.5 | 9 | 8 |
| 388 | Q14699 | Raftlin | RFTN1 | 33.4 | 15 | 15 |
| 389 | Q969V3 | Nicalin | NCLN | 24.7 | 13 | 13 |
| 390 | O43776 | Asparagine--tRNA ligase. cytoplasmic | NARS | 31.6 | 18 | 18 |
| 391 | O15270 | Serine palmitoyltransferase 2 | SPTLC2 | 12.3 | 6 | 6 |
| 392 | Q9NUL3 | Double-stranded RNA-binding protein Staufen homolog 2 | STAU2 | 17.5 | 8 | 8 |
| 393 | Q9UMX0 | Ubiquilin-1 | UBQLN1 | 31.1 | 10 | 5 |
| 394 | Q9Y5A9 | YTH domain-containing family protein 2 | YTHDF2 | 13.8 | 7 | 5 |
| 395 | Q9HA77 | Probable cysteine--tRNA ligase. mitochondrial | CARS2 | 16.3 | 8 | 8 |
| 396 | Q14194 | Dihydropyrimidinase-related protein 1 | CRMP1 | 29.5 | 10 | 10 |
| 397 | P15586 | N-acetylglucosamine-6-sulfatase | GNS | 15.6 | 8 | 8 |
| 398 | Q96S66 | Chloride channel CLIC-like protein 1 | CLCC1 | 24.5 | 10 | 10 |
| 399 | Q5NDL2 | EGF domain-specific O-linked N-acetylglucosamine transferase | EOGT | 16.1 | 9 | 9 |
| 400 | Q9Y5J1 | U3 small nucleolar RNA-associated protein 18 homolog | UTP18 | 18 | 8 | 8 |
| 401 | Q9BVP2 | Guanine nucleotide-binding protein-like 3 | GNL3 | 17.1 | 9 | 9 |
| 402 | O75879 | Glutamyl-tRNA(Gln) amidotransferase subunit B. mitochondrial | GATB | 11.3 | 7 | 7 |
| 403 | Q6ZWJ1 | Syntaxin-binding protein 4 | STXBP4 | 17.4 | 8 | 8 |
| 404 | Q96S52 | GPI transamidase component PIG-S | PIGS | 18.2 | 9 | 9 |
| 405 | O95817 | BAG family molecular chaperone regulator 3 | BAG3 | 11.5 | 5 | 5 |
| 406 | Q96L92 | Sorting nexin-27 | SNX27 | 15.7 | 8 | 8 |
| 407 | O14773 | Tripeptidyl-peptidase 1 | TPP1 | 10.7 | 5 | 5 |
| 408 | Q9H223 | EH domain-containing protein 4 | EHD4 | 16.6 | 7 | 6 |
| 409 | Q9NXV6 | CDKN2A-interacting protein | CDKN2AIP | 15.3 | 7 | 7 |
| 410 | Q9NZW5 | MAGUK p55 subfamily member 6 | MPP6 | 47.4 | 24 | 22 |
| 411 | Q9NPH2 | Inositol-3-phosphate synthase 1 | ISYNA1 | 23.5 | 11 | 11 |
| 412 | Q9P0J1 | [Pyruvate dehydrogenase [acetyl-transferring]]-phosphatase 1. mitochondrial | PDP1 | 13.4 | 7 | 7 |
| 413 | O43242 | 26S proteasome non-ATPase regulatory subunit 3 | PSMD3 | 39 | 19 | 19 |
| 414 | P33240 | Cleavage stimulation factor subunit 2 | CSTF2 | 25.6 | 5 | 5 |
| 415 | Q9BVL2 | Nucleoporin p58/p45 | NUP58 | 18.4 | 10 | 10 |
| 416 | P07947 | Tyrosine-protein kinase Yes | YES1 | 32.2 | 9 | 8 |
| 417 | Q9H857 | 5-nucleotidase domain-containing protein 2 | NT5DC2 | 11.9 | 7 | 7 |
| 418 | Q13153 | Serine/threonine-protein kinase PAK 1 | PAK1 | 25.5 | 5 | 3 |
| 419 | Q9H4M9 | EH domain-containing protein 1 | EHD1 | 23.6 | 11 | 7 |
| 420 | Q69YN2 | CWF19-like protein 1 | CWF19L1 | 13.9 | 6 | 6 |
| 421 | P35520 | Cystathionine beta-synthase | CBS | 16.9 | 9 | 9 |
| 422 | Q6P1J9 | Parafibromin | CDC73 | 13.2 | 6 | 6 |
| 423 | Q6UWP8 | Suprabasin | SBSN | 32.7 | 10 | 10 |
| 424 | Q9BYV6 | Tripartite motif-containing protein 55 | TRIM55 | 28.6 | 16 | 16 |
| 425 | Q9Y6I3 | Epsin-1 | EPN1 | 13.7 | 4 | 4 |
| 426 | P51687 | Sulfite oxidase. mitochondrial | SUOX | 22.6 | 12 | 12 |
| 427 | Q13564 | NEDD8-activating enzyme E1 regulatory subunit | NAE1 | 15.7 | 8 | 8 |
| 428 | Q02318 | Sterol 26-hydroxylase. mitochondrial | CYP27A1 | 10.7 | 6 | 6 |
| 429 | P52294 | Importin subunit alpha-5 | KPNA1 | 15.6 | 6 | 3 |
| 430 | Q8WVV9 | Heterogeneous nuclear ribonucleoprotein L-like | HNRNPLL | 46.7 | 19 | 19 |
| 431 | Q8N7H5 | RNA polymerase II-associated factor 1 homolog | PAF1 | 11.1 | 5 | 5 |
| 432 | P12931 | Proto-oncogene tyrosine-protein kinase Src | SRC | 34.1 | 16 | 11 |
| 433 | P04040 | Catalase | CAT | 34.5 | 18 | 18 |
| 434 | Q8NHP6 | Motile sperm domain-containing protein 2 | MOSPD2 | 10.2 | 6 | 6 |
| 435 | P04062 | Lysosomal acid glucosylceramidase | GBA | 23.5 | 11 | 11 |
| 436 | Q14554 | Protein disulfide-isomerase A5 | PDIA5 | 24.3 | 10 | 10 |
| 437 | Q9Y2X3 | Nucleolar protein 58 | NOP58 | 41.4 | 18 | 18 |
| 438 | Q9Y5Q8 | General transcription factor 3C polypeptide 5 | GTF3C5 | 17 | 7 | 7 |
| 439 | O15355 | Protein phosphatase 1G | PPM1G | 16.7 | 8 | 8 |
| 440 | P11413 | Glucose-6-phosphate 1-dehydrogenase | G6PD | 16.5 | 8 | 8 |
| 441 | Q9BTT6 | Leucine-rich repeat-containing protein 1 | LRRC1 | 43.5 | 12 | 12 |
| 442 | Q16625 | Occludin | OCLN | 10.2 | 5 | 5 |
| 443 | P54577 | Tyrosine--tRNA ligase. cytoplasmic | YARS | 33.3 | 18 | 18 |
| 444 | Q13596 | Sorting nexin-1 | SNX1 | 34.3 | 13 | 13 |
| 445 | Q99829 | Copine-1 | CPNE1 | 12.8 | 8 | 8 |
| 446 | Q9BZZ5 | Apoptosis inhibitor 5 | API5 | 45 | 27 | 27 |
| 447 | O43865 | S-adenosylhomocysteine hydrolase-like protein 1 | AHCYL1 | 20.2 | 12 | 5 |
| 448 | Q3LXA3 | Triokinase/FMN cyclase | TKFC | 13.9 | 6 | 6 |
| 449 | Q13356 | RING-type E3 ubiquitin-protein ligase PPIL2 | PPIL2 | 12.3 | 5 | 5 |
| 450 | Q5JPH6 | Probable glutamate--tRNA ligase. mitochondrial | EARS2 | 17.4 | 10 | 10 |
| 451 | Q08209 | Serine/threonine-protein phosphatase 2B catalytic subunit alpha isoform | PPP3CA | 18.8 | 9 | 6 |
| 452 | P07948 | Tyrosine-protein kinase Lyn | LYN | 22.1 | 9 | 9 |
| 453 | Q7Z4H8 | Protein O-glucosyltransferase 3 | POGLUT3 | 45.8 | 20 | 20 |
| 454 | O60749 | Sorting nexin-2 | SNX2 | 42 | 22 | 20 |
| 455 | O43172 | U4/U6 small nuclear ribonucleoprotein Prp4 | PRPF4 | 25.5 | 12 | 12 |
| 456 | O43278 | Kunitz-type protease inhibitor 1 | SPINT1 | 34.8 | 18 | 18 |
| 457 | P35269 | General transcription factor IIF subunit 1 | GTF2F1 | 11.4 | 4 | 4 |
| 458 | P48454 | Serine/threonine-protein phosphatase 2B catalytic subunit gamma isoform | PPP3CC | 12.3 | 4 | 4 |
| 459 | P07602 | Prosaposin | PSAP | 29.8 | 16 | 16 |
| 460 | Q13884 | Beta-1-syntrophin | SNTB1 | 17.1 | 8 | 8 |
| 461 | Q6UW63 | Protein O-glucosyltransferase 2 | POGLUT2 | 18.3 | 9 | 9 |
| 462 | Q13177 | Serine/threonine-protein kinase PAK 2 | PAK2 | 27.3 | 11 | 5 |
| 463 | O95747 | Serine/threonine-protein kinaseR1 | OXSR1 | 19 | 8 | 7 |
| 464 | Q13425 | Beta-2-syntrophin | SNTB2 | 14.4 | 7 | 7 |
| 465 | Q8TD30 | Alanine aminotransferase 2 | GPT2 | 38.2 | 17 | 17 |
| 466 | O00505 | Importin subunit alpha-4 | KPNA3 | 20 | 8 | 5 |
| 467 | Q9BZI7 | Regulator of nonsense transcripts 3B | UPF3B | 15.5 | 5 | 5 |
| 468 | Q92692 | Nectin-2 | NECTIN2 | 23.8 | 10 | 10 |
| 469 | O60832 | H/ACA ribonucleoprotein complex subunit DKC1 | DKC1 | 28 | 15 | 15 |
| 470 | Q13217 | DnaJ homolog subfamily C member 3 | DNAJC3 | 26 | 15 | 15 |
| 471 | Q9Y285 | Phenylalanine--tRNA ligase alpha subunit | FARSA | 18.5 | 8 | 8 |
| 472 | Q2TAY7 | WD40 repeat-containing protein SMU1 | SMU1 | 33.9 | 17 | 17 |
| 473 | Q9H0R6 | Glutamyl-tRNA(Gln) amidotransferase subunit mitochondrial | QRSL1 | 22.7 | 10 | 10 |
| 474 | P12081 | Histidine--tRNA ligase. cytoplasmic | HARS | 16.1 | 9 | 5 |
| 475 | Q06203 | Amidophosphoribosyltransferase | PPAT | 11.8 | 5 | 5 |
| 476 | P05186 | Alkaline phosphatase. tissue-nonspecific isozyme | ALPL | 13 | 5 | 5 |
| 477 | Q96RE7 | Nucleus accumbens-associated protein 1 | NACC1 | 12.7 | 5 | 5 |
| 478 | Q8WUA2 | Peptidyl-prolyl cis-trans isomerase-like 4 | PPIL4 | 17.3 | 6 | 6 |
| 479 | P51649 | Succinate-semialdehyde dehydrogenase. mitochondrial | ALDH5A1 | 39.8 | 18 | 18 |
| 480 | O43660 | Pleiotropic regulator 1 | PLRG1 | 21.2 | 11 | 11 |
| 481 | P14868 | Aspartate--tRNA ligase. cytoplasmic | DARS | 49.5 | 23 | 23 |
| 482 | Q9BXI6 | TBC1 domain family member 10A | TBC1D10A | 10.4 | 5 | 5 |
| 483 | Q96T60 | Bifunctional polynucleotide phosphatase/kinase | PNKP | 12.1 | 6 | 6 |
| 484 | Q9NQH7 | Xaa-Pro aminopeptidase 3 | XPNPEP3 | 16.2 | 7 | 7 |
| 485 | Q8NC56 | LEM domain-containing protein 2 | LEMD2 | 16.5 | 7 | 7 |
| 486 | Q96IF1 | LIM domain-containing protein ajuba | AJUBA | 16.7 | 7 | 7 |
| 487 | Q96JB5 | CDK5 regulatory subunit-associated protein 3 | CDK5RAP3 | 31 | 17 | 17 |
| 488 | P49590 | Probable histidine--tRNA ligase. mitochondrial | HARS2 | 15.6 | 4 | 4 |
| 489 | P53041 | Serine/threonine-protein phosphatase 5 | PPP5C | 12.6 | 6 | 6 |
| 490 | P30419 | Glycylpeptide N-tetradecanoyltransferase 1 | NMT1 | 19 | 9 | 9 |
| 491 | Q16850 | Lanosterol 14-alpha demethylase | CYP51A1 | 15.9 | 7 | 7 |
| 492 | Q92575 | UBX domain-containing protein 4 | UBXN4 | 27.8 | 10 | 10 |
| 493 | P51688 | N-sulphoglucosamine sulphohydrolase | SGSH | 26.7 | 10 | 10 |
| 494 | Q08170 | Serine/arginine-rich splicing factor 4 | SRSF4 | 25.7 | 18 | 14 |
| 495 | Q9NXG6 | Transmembrane prolyl 4-hydroxylase | P4HTM | 15.3 | 6 | 6 |
| 496 | P54868 | Hydroxymethylglutaryl-CoA synthase. mitochondrial | HMGCS2 | 20.5 | 10 | 9 |
| 497 | Q8NB12 | Histone-lysine N-methyltransferase SMYD1 | SMYD1 | 13.5 | 7 | 7 |
| 498 | Q9Y6G9 | Cytoplasmic dynein 1 light intermediate chain 1 | DYNC1LI1 | 13.4 | 5 | 5 |
| 499 | Q7Z434 | Mitochondrial antiviral-signaling protein | MAVS | 19.4 | 6 | 6 |
| 500 | Q99615 | DnaJ homolog subfamily C member 7 | DNAJC7 | 23.5 | 13 | 13 |
| 501 | P00390 | Glutathione reductase. mitochondrial | GSR | 16.3 | 7 | 7 |
| 502 | Q15172 | Serine/threonine-protein phosphatase 2A 56 kDa regulatory subunit alpha isoform | PPP2R5A | 11.5 | 5 | 5 |
| 503 | Q96F86 | Enhancer of mRNA-decapping protein 3 | EDC3 | 21.3 | 9 | 9 |
| 504 | P54578 | Ubiquitin carboxyl-terminal hydrolase 14 | USP14 | 31.6 | 14 | 14 |
| 505 | P02675 | Fibrinogen beta chain | FGB | 11.4 | 5 | 5 |
| 506 | Q9UI12 | V-type proton ATPase subunit H | ATP6V1H | 13.9 | 5 | 5 |
| 507 | Q96A33 | Coiled-coil domain-containing protein 47 | CCDC47 | 45.3 | 21 | 21 |
| 508 | Q92599 | Septin-8 | SEPTIN8 | 14.3 | 4 | 4 |
| 509 | P15170 | Eukaryotic peptide chain release factor GTP-binding subunit ERF3A | GSPT1 | 16 | 9 | 3 |
| 510 | Q9UNF0 | Protein kinase C and casein kinase substrate in neurons protein 2 | PACSIN2 | 33.7 | 16 | 15 |
| 511 | P61011 | Signal recognition particle 54 kDa protein | SRP54 | 25 | 14 | 14 |
| 512 | Q9BZK7 | F-box-like/WD repeat-containing protein TBL1XR1 | TBL1XR1 | 28.8 | 10 | 10 |
| 513 | Q13098 | COP9 signalosome complex subunit 1 | GPS1 | 15.1 | 7 | 7 |
| 514 | Q9NRY5 | Protein FAM114A2 | FAM114A2 | 23.4 | 9 | 9 |
| 515 | Q8WWY3 | U4/U6 small nuclear ribonucleoprotein Prp31 | PRPF31 | 20.4 | 11 | 11 |
| 516 | Q7Z3B4 | Nucleoporin p54 | NUP54 | 24.7 | 11 | 11 |
| 517 | Q92769 | Histone deacetylase 2 | HDAC2 | 36.3 | 13 | 10 |
| 518 | Q86WB0 | Nuclear-interacting partner of ALK | ZC3HC1 | 21.1 | 8 | 8 |
| 519 | Q13547 | Histone deacetylase 1 | HDAC1 | 33.2 | 8 | 8 |
| 520 | O60701 | UDP-glucose 6-dehydrogenase | UGDH | 17 | 6 | 6 |
| 521 | Q01650 | Large neutral amino acids transporter small subunit 1 | SLC7A5 | 13.6 | 7 | 7 |
| 522 | O76021 | Ribosomal L1 domain-containing protein 1 | RSL1D1 | 33.5 | 19 | 19 |
| 523 | P78324 | Tyrosine-protein phosphatase non-receptor type substrate 1 | SIRPA | 25.8 | 10 | 10 |
| 524 | P30566 | Adenylosuccinate lyase | ADSL | 17.1 | 9 | 9 |
| 525 | P00352 | Retinal dehydrogenase 1 | ALDH1A1 | 14.4 | 5 | 5 |
| 526 | Q96DZ1 | Endoplasmic reticulum lectin 1 | ERLEC1 | 12.2 | 5 | 5 |
| 527 | O43148 | mRNA cap guanine-N7 methyltransferase | RNMT | 28.6 | 15 | 15 |
| 528 | Q16537 | Serine/threonine-protein phosphatase 2A 56 kDa regulatory subunit epsilon isoform | PPP2R5E | 14.1 | 6 | 5 |
| 529 | Q75N03 | E3 ubiquitin-protein ligase Hakai | CBLL1 | 17.9 | 7 | 7 |
| 530 | P26196 | Probable ATP-dependent RNA helicase DDX6 | DDX6 | 29.6 | 13 | 13 |
| 531 | Q9NZM5 | Ribosome biogenesis protein NOP53 | NOP53 | 16.1 | 8 | 8 |
| 532 | Q9UHL4 | Dipeptidyl peptidase 2 | DPP7 | 18.5 | 8 | 8 |
| 533 | Q14108 | Lysosome membrane protein 2 | SCARB2 | 24.1 | 14 | 14 |
| 534 | O95319 | CUGBP Elav-like family member 2 | CELF2 | 19.9 | 6 | 6 |
| 535 | A1L020 | RNA-binding protein MEX3A | MEX3A | 33.7 | 12 | 11 |
| 536 | Q9H3G5 | Probable serine carboxypeptidase CPVL | CPVL | 11.6 | 5 | 5 |
| 537 | O00330 | Pyruvate dehydrogenase protein X component. mitochondrial | PDHX | 30.5 | 14 | 14 |
| 538 | Q9UN86 | Ras GTPase-activating protein-binding protein 2 | G3BP2 | 36.7 | 14 | 14 |
| 539 | O43237 | Cytoplasmic dynein 1 light intermediate chain 2 | DYNC1LI2 | 16.5 | 6 | 6 |
| 540 | Q96I59 | Probable asparagine--tRNA ligase. mitochondrial | NARS2 | 13 | 8 | 8 |
| 541 | Q6UX04 | Spliceosome-associated protein CWC27 homolog | CWC27 | 23.3 | 8 | 8 |
| 542 | P22570 | NADPH:adrenodoxin oxidoreductase. mitochondrial | FDXR | 14.9 | 8 | 8 |
| 543 | O43854 | EGF-like repeat and discoidin I-like domain-containing protein 3 | EDIL3 | 13.3 | 6 | 6 |
| 544 | P15289 | Arylsulfatase A | ARSA | 15.4 | 6 | 6 |
| 545 | Q9UBB4 | Ataxin-10 | ATXN10 | 16.2 | 6 | 6 |
| 546 | P11182 | Lipoamide acyltransferase component of branched-chain alpha-keto acid dehydrogenase complex. mitochondrial | DBT | 20.5 | 12 | 12 |
| 547 | Q6NUK1 | Calcium-binding mitochondrial carrier protein SCaMC-1 | SLC25A24 | 23.3 | 10 | 10 |
| 548 | Q16204 | Coiled-coil domain-containing protein 6 | CCDC6 | 13.7 | 7 | 7 |
| 549 | Q9ULV4 | Coronin-1C | CORO1C | 53.2 | 25 | 24 |
| 550 | P23381 | Tryptophan--tRNA ligase. cytoplasmic | WARS | 37.4 | 13 | 13 |
| 551 | Q12849 | G-rich sequence factor 1 | GRSF1 | 17.5 | 8 | 8 |
| 552 | P07099 | Epoxide hydrolase 1 | EPHX1 | 19.8 | 10 | 10 |
| 553 | P50579 | Methionine aminopeptidase 2 | METAP2 | 32.4 | 11 | 11 |
| 554 | Q96KP4 | Cytosolic non-specific dipeptidase | CNDP2 | 16.8 | 8 | 8 |
| 555 | P56182 | Ribosomal RNA processing protein 1 homolog A | RRP1 | 11.7 | 5 | 5 |
| 556 | P40123 | Adenylyl cyclase-associated protein 2 | CAP2 | 17.6 | 8 | 8 |
| 557 | O15269 | Serine palmitoyltransferase 1 | SPTLC1 | 32.8 | 15 | 15 |
| 558 | P20073 | Annexin A7 | ANXA7 | 10 | 5 | 5 |
| 559 | Q9BXK5 | Bcl-2-like protein 13 | BCL2L13 | 19.6 | 8 | 8 |
| 560 | Q96CS3 | FAS-associated factor 2 | FAF2 | 29.9 | 13 | 12 |
| 561 | Q13867 | Bleomycin hydrolase | BLMH | 18 | 8 | 8 |
| 562 | O75306 | NADH dehydrogenase [ubiquinone] iron-sulfur protein 2. mitochondrial | NDUFS2 | 16.8 | 7 | 7 |
| 563 | Q9ULA0 | Aspartyl aminopeptidase | DNPEP | 10.1 | 4 | 4 |
| 564 | Q92733 | Proline-rich protein PRCC | PRCC | 31.6 | 8 | 8 |
| 565 | P48637 | Glutathione synthetase | GSS | 13.3 | 7 | 7 |
| 566 | Q9UJW0 | Dynactin subunit 4 | DCTN4 | 16.5 | 6 | 6 |
| 567 | P60228 | Eukaryotic translation initiation factor 3 subunit E | EIF3E | 21.6 | 10 | 10 |
| 568 | Q92879 | CUGBP Elav-like family member 1 | CELF1 | 29.6 | 18 | 11 |
| 569 | P55081 | Microfibrillar-associated protein 1 | MFAP1 | 21.4 | 8 | 8 |
| 570 | P48723 | Heat shock 70 kDa protein 13 | HSPA13 | 14.6 | 6 | 6 |
| 571 | Q01518 | Adenylyl cyclase-associated protein 1 | CAP1 | 39.6 | 18 | 18 |
| 572 | Q96JJ7 | Protein disulfide-isomerase TMX3 | TMX3 | 16.5 | 6 | 6 |
| 573 | O43818 | U3 small nucleolar RNA-interacting protein 2 | RRP9 | 23.2 | 11 | 11 |
| 574 | Q02790 | Peptidyl-prolyl cis-trans isomerase FKBP4 | FKBP4 | 34.9 | 13 | 13 |
| 575 | Q53EL6 | Programmed cell death protein 4 | PDCD4 | 19.6 | 9 | 9 |
| 576 | O95232 | Luc7-like protein 3 | LUC7L3 | 16.2 | 7 | 7 |
| 577 | Q6YP21 | Kynurenine--oxoglutarate transaminase 3 | KYAT3 | 20.7 | 7 | 7 |
| 578 | O43615 | Mitochondrial import inner membrane translocase subunit TIM44 | TIMM44 | 37.2 | 18 | 18 |
| 579 | Q8NFQ8 | Torsin-1A-interacting protein 2 | TOR1AIP2 | 28.3 | 10 | 9 |
| 580 | Q8NFQ8 | Torsin-1A-interacting protein 2 | TOR1AIP2 | 28.3 | 10 | 9 |
| 581 | Q13451 | Peptidyl-prolyl cis-trans isomerase FKBP5 | FKBP5 | 25.4 | 11 | 11 |
| 582 | P41091 | Eukaryotic translation initiation factor 2 subunit 3 | EIF2S3 | 29 | 12 | 12 |
| 583 | O43493 | Trans-Golgi network integral membrane protein 2 | TGOLN2 | 30.5 | 13 | 13 |
| 584 | Q96GQ5 | RUS1 family protein C16orf58 | C16orf58 | 10.9 | 5 | 5 |
| 585 | P49821 | NADH dehydrogenase [ubiquinone] flavoprotein 1. mitochondrial | NDUFV1 | 20.9 | 8 | 8 |
| 586 | O43159 | Ribosomal RNA-processing protein 8 | RRP8 | 10.1 | 4 | 4 |
| 587 | P31150 | Rab GDP dissociation inhibitor alpha | GDI1 | 35.1 | 7 | 7 |
| 588 | Q9HD26 | Golgi-associated PDZ and coiled-coil motif-containing protein | GOPC | 10.6 | 5 | 5 |
| 589 | P12694 | 2-oxoisovalerate dehydrogenase subunit alph mitochondrial | BCKDHA | 35.5 | 12 | 12 |
| 590 | Q92791 | Endoplasmic reticulum protein SC65 | P3H4 | 18.5 | 8 | 8 |
| 591 | Q9NP92 | 39S ribosomal protein S30. mitochondrial | MRPS30 | 19.1 | 5 | 5 |
| 592 | Q9H9P8 | L-2-hydroxyglutarate dehydrogenase. mitochondrial | L2HGDH | 27.6 | 11 | 11 |
| 593 | P80303 | Nucleobindin-2 | NUCB2 | 16.7 | 7 | 7 |
| 594 | Q9Y697 | Cysteine desulfurase. mitochondrial | NFS1 | 19 | 8 | 8 |
| 595 | Q9UKX7 | Nuclear pore complex protein Nup50 | NUP50 | 45.3 | 16 | 16 |
| 596 | Q9Y2G5 | GDP-fucose protein O-fucosyltransferase 2 | POFUT2 | 17.9 | 9 | 9 |
| 597 | P13489 | Ribonuclease inhibitor | RNH1 | 11.9 | 4 | 4 |
| 598 | Q9Y4P3 | Transducin beta-like protein 2 | TBL2 | 27.1 | 13 | 13 |
| 599 | Q4G0N4 | NAD kinase 2. mitochondrial | NADK2 | 34.2 | 14 | 14 |
| 600 | Q9NVA2 | Septin-11 | SEPTIN11 | 28.9 | 12 | 5 |
| 601 | Q96QA5 | Gasdermin-A | GSDMA | 18.4 | 7 | 7 |
| 602 | Q9Y6E0 | Serine/threonine-protein kinase 24 | STK24 | 11.3 | 4 | 3 |
| 603 | Q8IV38 | Ankyrin repeat and MYND domain-containing protein 2 | ANKMY2 | 12.2 | 5 | 5 |
| 604 | Q15428 | Splicing factor 3A subunit 2 | SF3A2 | 31.7 | 12 | 12 |
| 605 | Q7L592 | Protein arginine methyltransferase NDUFAF7. mitochondrial | NDUFAF7 | 12.5 | 5 | 5 |
| 606 | P55010 | Eukaryotic translation initiation factor 5 | EIF5 | 30.4 | 13 | 13 |
| 607 | P62191 | 26S proteasome regulatory subunit 4 | PSMC1 | 26.4 | 12 | 11 |
| 608 | Q9BRK3 | Matrix remodeling-associated protein 8 | MXRA8 | 14.5 | 8 | 8 |
| 609 | P62495 | Eukaryotic peptide chain release factor subunit 1 | ETF1 | 13.7 | 4 | 4 |
| 610 | Q06265 | Exosome complex component RRP45 | EXOSC9 | 18.7 | 7 | 7 |
| 611 | P56545 | C-terminal-binding protein 2 | CTBP2 | 18 | 10 | 6 |
| 612 | O43464 | Serine protease HTRA2. mitochondrial | HTRA2 | 14.6 | 6 | 6 |
| 613 | P35998 | 26S proteasome regulatory subunit 7 | PSMC2 | 27.7 | 11 | 11 |
| 614 | Q8WU90 | Zinc finger CCCH domain-containing protein 15 | ZC3H15 | 15.5 | 7 | 7 |
| 615 | P42696 | RNA-binding protein 34 | RBM34 | 15.8 | 6 | 6 |
| 616 | Q9UKS6 | Protein kinase C and casein kinase substrate in neurons protein 3 | PACSIN3 | 11.8 | 5 | 5 |
| 617 | Q05048 | Cleavage stimulation factor subunit 1 | CSTF1 | 23 | 10 | 10 |
| 618 | P11117 | Lysosomal acid phosphatase | ACP2 | 18 | 8 | 8 |
| 619 | Q9Y6E2 | Basic leucine zipper and W2 domain-containing protein 2 | BZW2 | 28.6 | 14 | 14 |
| 620 | Q92947 | Glutaryl-CoA dehydrogenase. mitochondrial | GCDH | 14.2 | 6 | 6 |
| 621 | Q9BZE1 | 39S ribosomal protein L37. mitochondrial | MRPL37 | 17.5 | 7 | 7 |
| 622 | Q7L1Q6 | Basic leucine zipper and W2 domain-containing protein 1 | BZW1 | 36.8 | 14 | 14 |
| 623 | P82675 | 28S ribosomal protein S5. mitochondrial | MRPS5 | 12.3 | 5 | 5 |
| 624 | Q9UGI8 | Testin | TES | 12.8 | 6 | 6 |
| 625 | P22830 | Ferrochelatase. mitochondrial | FECH | 15.1 | 6 | 6 |
| 626 | P61812 | Transforming growth factor beta-2 proprotein | TGFB2 | 15.2 | 7 | 7 |
| 627 | Q9C0E8 | Endoplasmic reticulum junction formation protein lunapark | LNPK | 26.4 | 9 | 9 |
| 628 | P23526 | Adenosylhomocysteinase | AHCY | 38.2 | 17 | 17 |
| 629 | Q9GZL7 | Ribosome biogenesis protein WDR12 | WDR12 | 14.2 | 4 | 4 |
| 630 | Q92552 | 28S ribosomal protein S27. mitochondrial | MRPS27 | 18.4 | 6 | 6 |
| 631 | P09543 | 2.3-cyclic-nucleotide 3-phosphodiesterase | CNP | 30.4 | 11 | 11 |
| 632 | Q13363 | C-terminal-binding protein 1 | CTBP1 | 17 | 4 | 4 |
| 633 | P45954 | Short/branched chain specific acyl-CoA dehydrogenase. mitochondrial | ACADSB | 37.7 | 13 | 13 |
| 634 | Q9Y606 | tRNA pseudouridine synthase A | PUS1 | 12.6 | 5 | 5 |
| 635 | P43686 | 26S proteasome regulatory subunit 6B | PSMC4 | 17 | 5 | 5 |
| 636 | O75955 | Flotillin-1 | FLOT1 | 10.8 | 5 | 5 |
| 637 | Q7L0Y3 | tRNA methyltransferase 10 homolog C | TRMT10C | 28.8 | 11 | 11 |
| 638 | P09104 | Gamma-enolase | ENO2 | 23.5 | 5 | 4 |
| 639 | Q8NBX0 | Saccharopine dehydrogenase-like oxidoreductase | SCCPDH | 21 | 6 | 6 |
| 640 | Q9H8Y8 | Golgi reassembly-stacking protein 2 | GORASP2 | 29.4 | 10 | 9 |
| 641 | P22234 | Multifunctional protein ADE2 | PAICS | 22.8 | 11 | 11 |
| 642 | O60664 | Perilipin-3 | PLIN3 | 25.8 | 10 | 10 |
| 643 | Q14254 | Flotillin-2 | FLOT2 | 23.4 | 10 | 10 |
| 644 | Q15120 | [Pyruvate dehydrogenase (acetyl-transferring)] kinase isozyme 3. mitochondrial | PDK3 | 12.6 | 4 | 4 |
| 645 | Q15018 | BRISC complex subunit Abraxas 2 | ABRAXAS2 | 14.7 | 6 | 6 |
| 646 | Q9NW64 | Pre-mRNA-splicing factor RBM22 | RBM22 | 11.7 | 5 | 5 |
| 647 | Q9Y5X3 | Sorting nexin-5 | SNX5 | 11.9 | 4 | 4 |
| 648 | P49841 | Glycogen synthase kinase-3 beta | GSK3B | 17.4 | 6 | 4 |
| 649 | O75874 | Isocitrate dehydrogenase [NADP] cytoplasmic | IDH1 | 36 | 13 | 13 |
| 650 | O75718 | Cartilage-associated protein | CRTAP | 27.2 | 10 | 10 |
| 651 | Q9Y383 | Putative RNA-binding protein Luc7-like 2 | LUC7L2 | 11.2 | 4 | 2 |
| 652 | O14874 | [3-methyl-2-oxobutanoate dehydrogenase [lipoamide]] kinase. mitochondrial | BCKDK | 12.4 | 4 | 4 |
| 653 | Q8IWB7 | WD repeat and FYVE domain-containing protein 1 | WDFY1 | 18.5 | 7 | 7 |
| 654 | Q9BXW7 | Haloacid dehalogenase-like hydrolase domain-containing 5 | HDHD5 | 29.3 | 11 | 11 |
| 655 | P31323 | cAMP-dependent protein kinase type II-beta regulatory subunit | PRKAR2B | 32.3 | 9 | 9 |
| 656 | Q96P63 | Serpin B12 | SERPINB12 | 13.8 | 8 | 8 |
| 657 | Q9BT78 | COP9 signalosome complex subunit 4 | COPS4 | 10.1 | 4 | 4 |
| 658 | P17174 | Aspartate aminotransferase. cytoplasmic | GOT1 | 12.3 | 5 | 5 |
| 659 | P48426 | Phosphatidylinositol 5-phosphate 4-kinase type-2 alpha | PIP4K2A | 16 | 6 | 4 |
| 660 | Q9BY77 | Polymerase delta-interacting protein 3 | POLDIP3 | 25.9 | 10 | 10 |
| 661 | Q99442 | Translocation protein SEC62 | SEC62 | 13.5 | 7 | 7 |
| 662 | P82933 | 28S ribosomal protein S9. mitochondrial | MRPS9 | 31.1 | 13 | 13 |
| 663 | P62195 | 26S proteasome regulatory subunit 8 | PSMC5 | 22.4 | 7 | 6 |
| 664 | P51398 | 28S ribosomal protein S29. mitochondrial | DAP3 | 19.1 | 6 | 6 |
| 665 | Q96HD1 | Cysteine-rich with EGF-like domain protein 1 | CRELD1 | 17.1 | 6 | 6 |
| 666 | Q8NBJ4 | Golgi membrane protein 1 | GOLM1 | 20.7 | 7 | 7 |
| 667 | P15151 | Poliovirus receptor | PVR | 14.6 | 4 | 4 |
| 668 | P24752 | Acetyl-CoA acetyltransferase. mitochondrial | ACAT1 | 41 | 13 | 13 |
| 669 | P08138 | Tumor necrosis factor receptor superfamily member 16 | NGFR | 11.9 | 4 | 4 |
| 670 | Q8N2K0 | Lysophosphatidylserine lipase ABHD12 | ABHD12 | 15.3 | 5 | 5 |
| 671 | Q96I25 | Splicing factor 45 | RBM17 | 20.4 | 7 | 7 |
| 672 | P31689 | DnaJ homolog subfamily A member 1 | DNAJA1 | 18.1 | 7 | 7 |
| 673 | P61160 | Actin-related protein 2 | ACTR2 | 15.2 | 6 | 6 |
| 674 | Q9NTK5 | Obg-like ATPase 1 | OLA1 | 17.7 | 6 | 6 |
| 675 | Q8N6T3 | ADP-ribosylation factor GTPase-activating protein 1 | ARFGAP1 | 14.3 | 5 | 5 |
| 676 | Q96DV4 | 39S ribosomal protein L38. mitochondrial | MRPL38 | 20.8 | 6 | 6 |
| 677 | P29508 | Serpin B3 | SERPINB3 | 37.7 | 13 | 13 |
| 678 | P29558 | RNA-binding motif. single-stranded-interacting protein 1 | RBMS1 | 10.1 | 4 | 4 |
| 679 | Q16543 | Hsp90 co-chaperone Cdc37 | CDC37 | 26.5 | 9 | 9 |
| 680 | Q9Y639 | Neuroplastin | NPTN | 26.4 | 11 | 11 |
| 681 | P16219 | Short-chain specific acyl-CoA dehydrogenase. mitochondrial | ACADS | 47.8 | 14 | 14 |
| 682 | O15382 | Branched-chain-amino-acid aminotransferase. mitochondrial | BCAT2 | 37.5 | 9 | 9 |
| 683 | Q15007 | Pre-mRNA-splicing regulator WTAP | WTAP | 28.5 | 9 | 9 |
| 684 | Q13561 | Dynactin subunit 2 | DCTN2 | 15.2 | 6 | 6 |
| 685 | P62333 | 26S proteasome regulatory subunit 10B | PSMC6 | 18.8 | 7 | 7 |
| 686 | Q9NR46 | Endophilin-B2 | SH3GLB2 | 11.6 | 4 | 4 |
| 687 | P48728 | Aminomethyltransferase. mitochondrial | AMT | 11.4 | 5 | 5 |
| 688 | Q99447 | Ethanolamine-phosphate cytidylyltransferase | PCYT2 | 11.6 | 4 | 4 |
| 689 | Q8NBI6 | Xyloside xylosyltransferase 1 | XXYLT1 | 10.9 | 4 | 4 |
| 690 | Q9UQ80 | Proliferation-associated protein 2G4 | PA2G4 | 34.8 | 13 | 13 |
| 691 | Q13601 | KRR1 small subunit processome component homolog | KRR1 | 16 | 6 | 6 |
| 692 | Q9NX58 | Cell growth-regulating nucleolar protein | LYAR | 16.1 | 6 | 6 |
| 693 | Q53HC9 | EARP and GARP complex-interacting protein 1 | EIPR1 | 10.1 | 4 | 4 |
| 694 | O75521 | Enoyl-CoA delta isomerase 2. mitochondrial | ECI2 | 12.4 | 6 | 6 |
| 695 | Q9NUD5 | Zinc finger CCHC domain-containing protein 3 | ZCCHC3 | 10.7 | 5 | 5 |
| 696 | Q6NVY1 | 3-hydroxyisobutyryl-CoA hydrolase. mitochondrial | HIBCH | 18.4 | 8 | 8 |
| 697 | Q6NZI2 | Caveolae-associated protein 1 | CAVIN1 | 14.4 | 5 | 5 |
| 698 | Q92734 | Protein TFG | TFG | 12 | 4 | 4 |
| 699 | Q9BRX2 | Protein pelota homolog | PELO | 16.1 | 6 | 6 |
| 700 | Q9Y282 | Endoplasmic reticulum-Golgi intermediate compartment protein 3 | ERGIC3 | 16.4 | 8 | 8 |
| 701 | P53582 | Methionine aminopeptidase 1 | METAP1 | 14 | 5 | 5 |
| 702 | P54727 | UV excision repair protein RAD23 homolog B | RAD23B | 21.3 | 7 | 7 |
| 703 | P21953 | 2-oxoisovalerate dehydrogenase subunit bet mitochondrial | BCKDHB | 28.8 | 7 | 7 |
| 704 | P10644 | cAMP-dependent protein kinase type I-alpha regulatory subunit | PRKAR1A | 10 | 5 | 5 |
| 705 | Q9UNM6 | 26S proteasome non-ATPase regulatory subunit 13 | PSMD13 | 13.8 | 6 | 6 |
| 706 | Q9NXS2 | Glutaminyl-peptide cyclotransferase-like protein | QPCTL | 12.3 | 5 | 5 |
| 707 | Q9H0C8 | Integrin-linked kinase-associated serine/threonine phosphatase 2C | ILKAP | 17.1 | 7 | 7 |
| 708 | Q99733 | Nucleosome assembly protein 1-like 4 | NAP1L4 | 17.1 | 5 | 4 |
| 709 | Q8NFZ8 | Cell adhesion molecule 4 | CADM4 | 25.5 | 7 | 7 |
| 710 | P61163 | Alpha-centractin | ACTR1A | 33 | 10 | 5 |
| 711 | P50453 | Serpin B9 | SERPINB9 | 20.7 | 8 | 8 |
| 712 | P41221 | Protein Wnt-5a | WNT5A | 10.8 | 4 | 4 |
| 713 | O43837 | Isocitrate dehydrogenase [NAD] subunit bet mitochondrial | IDH3B | 16.6 | 5 | 5 |
| 714 | P50148 | Guanine nucleotide-binding protein G(q) subunit alpha | GNAQ | 30.6 | 10 | 7 |
| 715 | Q96E39 | RNA binding motif protein. X-linked-like-1 | RBMXL1 | 56.4 | 5 | 5 |
| 716 | O75781 | Paralemmin-1 | PALM | 42.1 | 16 | 14 |
| 717 | Q9Y2S7 | Polymerase delta-interacting protein 2 | POLDIP2 | 29.1 | 8 | 8 |
| 718 | Q5T280 | Putative methyltransferase C9orf114 | SPOUT1 | 14.1 | 5 | 5 |
| 719 | Q99536 | Synaptic vesicle membrane protein VAT-1 homolog | VAT1 | 33.8 | 9 | 9 |
| 720 | Q15738 | Sterol-4-alpha-carboxylate 3-dehydrogenase. decarboxylating | NSDHL | 33 | 10 | 10 |
| 721 | Q9BRK5 | 45 kDa calcium-binding protein | SDF4 | 11.6 | 5 | 5 |
| 722 | Q15019 | Septin-2 | SEPTIN2 | 37.4 | 11 | 11 |
| 723 | P30533 | Alpha-2-macroglobulin receptor-associated protein | LRPAP1 | 19.6 | 8 | 8 |
| 724 | P08174 | Complement decay-accelerating factor | CD55 | 10.5 | 5 | 5 |
| 725 | P28482 | Mitogen-activated protein kinase 1 | MAPK1 | 17.5 | 5 | 4 |
| 726 | O43251 | RNA binding protein fox-1 homolog 2 | RBFOX2 | 13.3 | 5 | 5 |
| 727 | P50502 | Hsc70-interacting protein | ST13 | 19.2 | 6 | 6 |
| 728 | Q15050 | Ribosome biogenesis regulatory protein homolog | RRS1 | 18.9 | 7 | 7 |
| 729 | P78406 | mRNA export factor | RAE1 | 13.3 | 4 | 4 |
| 730 | Q9Y371 | Endophilin-B1 | SH3GLB1 | 23.6 | 7 | 6 |
| 731 | O95299 | NADH dehydrogenase [ubiquinone] 1 alpha subcomplex subunit 10. mitochondrial | NDUFA10 | 17.2 | 5 | 5 |
| 732 | Q9UNZ2 | NSFL1 cofactor p47 | NSFL1C | 15.9 | 5 | 5 |
| 733 | Q9UBS4 | DnaJ homolog subfamily B member 11 | DNAJB11 | 20.9 | 7 | 7 |
| 734 | Q9Y617 | Phosphoserine aminotransferase | PSAT1 | 12.7 | 4 | 4 |
| 735 | P61421 | V-type proton ATPase subunit d 1 | ATP6V0D1 | 12 | 4 | 4 |
| 736 | Q9BWF3 | RNA-binding protein 4 | RBM4 | 39 | 10 | 4 |
| 737 | Q12907 | Vesicular integral-membrane protein VIP36 | LMAN2 | 21.6 | 8 | 8 |
| 738 | P78310 | Coxsackievirus and adenovirus receptor | CXADR | 24.7 | 10 | 10 |
| 739 | O15372 | Eukaryotic translation initiation factor 3 subunit H | EIF3H | 22.7 | 9 | 9 |
| 740 | Q3ZCQ8 | Mitochondrial import inner membrane translocase subunit TIM50 | TIMM50 | 28.9 | 10 | 10 |
| 741 | Q9NYL9 | Tropomodulin-3 | TMOD3 | 13.6 | 5 | 4 |
| 742 | O43488 | Aflatoxin B1 aldehyde reductase member 2 | AKR7A2 | 14.2 | 4 | 4 |
| 743 | P09972 | Fructose-bisphosphate aldolase C | ALDOC | 42.6 | 9 | 9 |
| 744 | Q96DI7 | U5 small nuclear ribonucleoprotein 40 kDa protein | SNRNP40 | 35 | 8 | 8 |
| 745 | O75477 | Erlin-1 | ERLIN1 | 27.3 | 6 | 6 |
| 746 | Q4G0F5 | Vacuolar protein sorting-associated protein 26B | VPS26B | 15.2 | 4 | 4 |
| 747 | O95210 | Starch-binding domain-containing protein 1 | STBD1 | 17.6 | 5 | 5 |
| 748 | Q9NX62 | Inositol monophosphatase 3 | IMPAD1 | 16.7 | 4 | 4 |
| 749 | Q9P2W9 | Syntaxin-18 | STX18 | 12.5 | 4 | 4 |
| 750 | Q12972 | Nuclear inhibitor of protein phosphatase 1 | PPP1R8 | 20.2 | 5 | 5 |
| 751 | Q9UBE0 | SUMO-activating enzyme subunit 1 | SAE1 | 12.7 | 5 | 5 |
| 752 | Q9Y3F4 | Serine-threonine kinase receptor-associated protein | STRAP | 32.9 | 7 | 7 |
| 753 | P20042 | Eukaryotic translation initiation factor 2 subunit 2 | EIF2S2 | 18.3 | 8 | 8 |
| 754 | P98172 | Ephrin-B1 | EFNB1 | 23.7 | 5 | 5 |
| 755 | O15126 | Secretory carrier-associated membrane protein 1 | SCAMP1 | 24 | 6 | 6 |
| 756 | Q9H2U2 | Inorganic pyrophosphatase 2. mitochondrial | PPA2 | 37.4 | 11 | 11 |
| 757 | O94905 | Erlin-2 | ERLIN2 | 34.8 | 14 | 8 |
| 758 | P07858 | Cathepsin B | CTSB | 19.8 | 6 | 6 |
| 759 | Q96PU8 | Protein quaking | QKI | 29.9 | 9 | 9 |
| 760 | P01876 | Immunoglobulin heavy constant alpha 1 | IGHA1 | 22.4 | 7 | 4 |
| 761 | Q13449 | Limbic system-associated membrane protein | LSAMP | 14.2 | 5 | 5 |
| 762 | O60911 | Cathepsin L2 | CTSV | 11.1 | 4 | 4 |
| 763 | P62140 | Serine/threonine-protein phosphatase PP1-beta catalytic subunit | PPP1CB | 43.4 | 13 | 4 |
| 764 | O43684 | Mitotic checkpoint protein BUB3 | BUB3 | 25.9 | 8 | 8 |
| 765 | P51665 | 26S proteasome non-ATPase regulatory subunit 7 | PSMD7 | 21.6 | 5 | 5 |
| 766 | Q14257 | Reticulocalbin-2 | RCN2 | 35 | 9 | 9 |
| 767 | Q9HBH5 | Retinol dehydrogenase 14 | RDH14 | 13.4 | 4 | 4 |
| 768 | P61964 | WD repeat-containing protein 5 | WDR5 | 14.4 | 4 | 4 |
| 769 | Q13347 | Eukaryotic translation initiation factor 3 subunit I | EIF3I | 37.2 | 8 | 8 |
| 770 | Q15417 | Calponin-3 | CNN3 | 18.8 | 6 | 6 |
| 771 | P05198 | Eukaryotic translation initiation factor 2 subunit 1 | EIF2S1 | 17.8 | 6 | 6 |
| 772 | O00151 | PDZ and LIM domain protein 1 | PDLIM1 | 16.1 | 4 | 4 |
| 773 | Q16698 | 2.4-dienoyl-CoA reductase. mitochondrial | DECR1 | 25.1 | 7 | 7 |
| 774 | P30519 | Heme oxygenase 2 | HMOX2 | 17.7 | 5 | 5 |
| 775 | P45379 | Troponin T. cardiac muscle | TNNT2 | 41.6 | 15 | 15 |
| 776 | Q96CX2 | BTB/POZ domain-containing protein KCTD12 | KCTD12 | 29.5 | 11 | 11 |
| 777 | O75821 | Eukaryotic translation initiation factor 3 subunit G | EIF3G | 23.1 | 7 | 7 |
| 778 | Q96HY6 | DDRGK domain-containing protein 1 | DDRGK1 | 15.6 | 4 | 4 |
| 779 | P62714 | Serine/threonine-protein phosphatase 2A catalytic subunit beta isoform | PPP2CB | 16.2 | 4 | 4 |
| 780 | P27695 | DNA-(apurinic or apyrimidinic site) lyase | APEX1 | 17 | 4 | 4 |
| 781 | P55735 | Protein SEC13 homolog | SEC13 | 14.6 | 4 | 4 |
| 782 | Q8TC12 | Retinol dehydrogenase 11 | RDH11 | 24.5 | 6 | 6 |
| 783 | Q9BXY0 | Protein MAK16 homolog | MAK16 | 12.3 | 4 | 4 |
| 784 | Q9BRJ2 | 39S ribosomal protein L45. mitochondrial | MRPL45 | 15.4 | 5 | 5 |
| 785 | Q08257 | Quinone oxidoreductase | CRYZ | 21.3 | 5 | 5 |
| 786 | P63244 | Receptor of activated protein C kinase 1 | RACK1 | 64.7 | 18 | 18 |
| 787 | Q12841 | Follistatin-related protein 1 | FSTL1 | 13.6 | 5 | 5 |
| 788 | P16422 | Epithelial cell adhesion molecule | EPCAM | 33.1 | 10 | 10 |
| 789 | Q96AG4 | Leucine-rich repeat-containing protein 59 | LRRC59 | 51.8 | 15 | 15 |
| 790 | Q00577 | Transcriptional activator protein Pur-alpha | PURA | 15.8 | 6 | 5 |
| 791 | Q9HC38 | Glyoxalase domain-containing protein 4 | GLOD4 | 16.3 | 5 | 5 |
| 792 | Q8NFV4 | Protein ABHD11 | ABHD11 | 25.4 | 7 | 7 |
| 793 | Q96GK7 | Fumarylacetoacetate hydrolase domain-containing protein 2A | FAHD2A | 15 | 4 | 4 |
| 794 | Q92890 | Ubiquitin recognition factor in ER-associated degradation protein 1 | UFD1 | 14.7 | 4 | 4 |
| 795 | Q96KN1 | Protein LRATD2 | LRATD2 | 25.8 | 7 | 7 |
| 796 | P35914 | Hydroxymethylglutaryl-CoA lyase. mitochondrial | HMGCL | 24 | 7 | 7 |
| 797 | O15144 | Actin-related protein 2/3 complex subunit 2 | ARPC2 | 22 | 5 | 5 |
| 798 | Q53GQ0 | Very-long-chain 3-oxoacyl-CoA reductase | HSD17B12 | 27.2 | 7 | 7 |
| 799 | P25311 | Zinc-alpha-2-glycoprotein | AZGP1 | 44.3 | 11 | 11 |
| 800 | P00387 | NADH-cytochrome b5 reductase 3 | CYB5R3 | 49.8 | 11 | 11 |
| 801 | Q9UBR2 | Cathepsin Z | CTSZ | 10.2 | 4 | 4 |
| 802 | Q8NBJ7 | Inactive C-alpha-formylglycine-generating enzyme 2 | SUMF2 | 19.9 | 5 | 5 |
| 803 | Q9H4A6 | Golgi phosphoprotein 3 | GOLPH3 | 16.1 | 4 | 4 |
| 804 | Q99439 | Calponin-2 | CNN2 | 25.2 | 8 | 7 |
| 805 | Q16762 | Thiosulfate sulfurtransferase | TST | 28.6 | 6 | 6 |
| 806 | Q04323 | UBX domain-containing protein 1 | UBXN1 | 24.6 | 6 | 6 |
| 807 | Q9BPW8 | Protein NipSnap homolog 1 | NIPSNAP1 | 36.6 | 10 | 9 |
| 808 | Q9Y399 | 28S ribosomal protein S2. mitochondrial | MRPS2 | 14.9 | 5 | 5 |
| 809 | Q96QR8 | Transcriptional activator protein Pur-beta | PURB | 16.7 | 4 | 4 |
| 810 | P25325 | 3-mercaptopyruvate sulfurtransferase | MPST | 27.9 | 7 | 7 |
| 811 | P06753 | Tropomyosin alpha-3 chain | TPM3 | 41.8 | 6 | 3 |
| 812 | P52907 | F-actin-capping protein subunit alpha-1 | CAPZA1 | 28.7 | 6 | 5 |
| 813 | Q969X5 | Endoplasmic reticulum-Golgi intermediate compartment protein 1 | ERGIC1 | 13.1 | 4 | 4 |
| 814 | Q86WA6 | Valacyclovir hydrolase | BPHL | 28.2 | 4 | 4 |
| 815 | Q9H1Y0 | Autophagy protein 5 | ATG5 | 17.1 | 4 | 4 |
| 816 | Q96CN7 | Isochorismatase domain-containing protein 1 | ISOC1 | 34.6 | 8 | 8 |
| 817 | Q14192 | Four and a half LIM domains protein 2 | FHL2 | 14.7 | 4 | 4 |
| 818 | Q9H3N1 | Thioredoxin-related transmembrane protein 1 | TMX1 | 16.1 | 5 | 5 |
| 819 | P27105 | Erythrocyte band 7 integral membrane protein | STOM | 46.5 | 11 | 11 |
| 820 | Q9H2W6 | 39S ribosomal protein L46. mitochondrial | MRPL46 | 15.8 | 5 | 5 |
| 821 | Q86Y82 | Syntaxin-12 | STX12 | 14.9 | 4 | 4 |
| 822 | P21912 | Succinate dehydrogenase [ubiquinone] iron-sulfur subunit. mitochondrial | SDHB | 21.4 | 6 | 6 |
| 823 | O75494 | Serine/arginine-rich splicing factor 10 | SRSF10 | 22.5 | 5 | 5 |
| 824 | P29692 | Elongation factor 1-delta | EEF1D | 22.1 | 4 | 4 |
| 825 | P20645 | Cation-dependent mannose-6-phosphate receptor | M6PR | 19.1 | 6 | 6 |
| 826 | Q6ZVX7 | F-box only protein 50 | NCCRP1 | 15.6 | 4 | 4 |
| 827 | P21796 | Voltage-dependent anion-selective channel protein 1 | VDAC1 | 45.6 | 11 | 11 |
| 828 | P53701 | Cytochrome c-type heme lyase | HCCS | 13.4 | 4 | 4 |
| 829 | Q15691 | Microtubule-associated protein RP/EB family member 1 | MAPRE1 | 40.3 | 6 | 6 |
| 830 | O75937 | DnaJ homolog subfamily C member 8 | DNAJC8 | 34.4 | 8 | 8 |
| 831 | P35232 | Prohibitin | PHB | 43.4 | 10 | 10 |
| 832 | P25786 | Proteasome subunit alpha type-1 | PSMA1 | 24 | 5 | 5 |
| 833 | Q9HD33 | 39S ribosomal protein L47. mitochondrial | MRPL47 | 19.2 | 4 | 4 |
| 834 | P24539 | ATP synthase F(0) complex subunit B1. mitochondrial | ATP5PB | 34.8 | 8 | 8 |
| 835 | Q9Y696 | Chloride intracellular channel protein 4 | CLIC4 | 30 | 4 | 4 |
| 836 | Q9UFN0 | Protein NipSnap homolog 3A | NIPSNAP3A | 22.3 | 4 | 4 |
| 837 | P09661 | U2 small nuclear ribonucleoprotein A | SNRPA1 | 25.1 | 5 | 5 |
| 838 | P61981 | 14-3-3 protein gamma | YWHAG | 25.1 | 4 | 4 |
| 839 | Q9P0L0 | Vesicle-associated membrane protein-associated protein A | VAPA | 22.9 | 5 | 4 |
| 840 | P0DN76 | Splicing factor U2AF 35 kDa subunit-like protein | U2AF1L5 | 35.8 | 7 | 7 |
| 841 | P60900 | Proteasome subunit alpha type-6 | PSMA6 | 23.6 | 4 | 4 |
| 842 | Q9BVK6 | Transmembrane emp24 domain-containing protein 9 | TMED9 | 22.1 | 6 | 4 |
| 843 | Q99714 | 3-hydroxyacyl-CoA dehydrogenase type-2 | HSD17B10 | 78.9 | 14 | 14 |
| 844 | P51858 | Hepatoma-derived growth factor | HDGF | 23.8 | 5 | 5 |
| 845 | O43809 | Cleavage and polyadenylation specificity factor subunit 5 | NUDT21 | 37.9 | 5 | 5 |
| 846 | Q9BRX8 | Peroxiredoxin-like 2A | PRXL2A | 35.4 | 8 | 8 |
| 847 | Q9UIJ7 | GTP:AMP phosphotransferase AK3. mitochondrial | AK3 | 23.8 | 5 | 5 |
| 848 | P27144 | Adenylate kinase 4. mitochondrial | AK4 | 52.9 | 9 | 9 |
| 849 | P62906 | 60S ribosomal protein L10a | RPL10A | 24.4 | 5 | 5 |
| 850 | P62491 | Ras-related protein Rab-11A | RAB11A | 31.5 | 7 | 6 |
| 851 | P61106 | Ras-related protein Rab-14 | RAB14 | 47.9 | 8 | 8 |
| 852 | P23284 | Peptidyl-prolyl cis-trans isomerase B | PPIB | 38.4 | 8 | 8 |
| 853 | P82979 | SAP domain-containing ribonucleoprotein | SARNP | 22.9 | 4 | 4 |
| 854 | P40429 | 60S ribosomal protein L13a | RPL13A | 34.5 | 10 | 10 |
| 855 | P61019 | Ras-related protein Rab-2A | RAB2A | 61.8 | 12 | 5 |
| 856 | P51148 | Ras-related protein Rab-5C | RAB5C | 32.9 | 5 | 5 |
| 857 | P50914 | 60S ribosomal protein L14 | RPL14 | 18.1 | 4 | 4 |
| 858 | P09211 | Glutathione S-transferase P | GSTP1 | 23.3 | 4 | 4 |
| 859 | P48047 | ATP synthase subunit O. mitochondrial | ATP5PO | 25.8 | 4 | 4 |
| 860 | P46782 | 40S ribosomal protein S5 | RPS5 | 26.5 | 7 | 7 |
| 861 | P04792 | Heat shock protein beta-1 | HSPB1 | 55.6 | 7 | 7 |
| 862 | P80723 | Brain acid soluble protein 1 | BASP1 | 75.3 | 9 | 9 |
| 863 | P61586 | Transforming protein RhoA | RHOA | 47.2 | 8 | 8 |
| 864 | P63000 | Ras-related C3 botulinum toxin substrate 1 | RAC1 | 38 | 7 | 7 |
| 865 | Q02543 | 60S ribosomal protein L18a | RPL18A | 45.5 | 8 | 8 |
| 866 | P51571 | Translocon-associated protein subunit delta | SSR4 | 31.2 | 5 | 5 |
| 867 | P62280 | 40S ribosomal protein S11 | RPS11 | 62 | 13 | 13 |
| 868 | P62277 | 40S ribosomal protein S13 | RPS13 | 48.3 | 8 | 8 |
| 869 | P61353 | 60S ribosomal protein L27 | RPL27 | 38.2 | 4 | 4 |
